# A Universal Nucleoside‐to‐Nucleoside‐5′‐Triphosphate Enzyme Cascade Driven by Polyphosphate

**DOI:** 10.1002/anie.6720227

**Published:** 2026-06-11

**Authors:** Jonathan P. Suess, Nicolas V. Cornelissen

**Affiliations:** ^1^ Institute of Biochemistry University of Münster Münster Germany

**Keywords:** biocatalysis, enzyme cascade, modified nucleotides, nucleoside‐5′‐triphosphate, nucleoside‐to‐NTP

## Abstract

Nucleoside‐5′‐triphosphates (NTPs) are important building blocks for nucleic acids, but the efficiency of their chemical synthesis is limited by the low yield of the final 5′‐triphosphorylation reaction. Here, we present an enzymatic cascade reaction involving just two enzymes that enable nucleosides‐to‐NTPs conversion for all canonical rNTPs and dNTPs. The cascade is used in a single one‐pot operation driven by cost‐efficient sodium polyphosphate (polyP). The broad‐spectrum deoxynucleoside kinase (Dm‐dNK) from *Drosophila melanogaster* converts nucleosides into nucleoside‐5′‐monophosphates (NMPs) using ATP. The polyphosphate kinase family 2, class III (EbPPK) from an *Erysipelotrichaceae* bacterium simultaneously recycles ATP required by Dm‐dNK and converts the NMPs to NTPs in a polyP‐dependent reaction. Importantly, only trace amounts of catalytic ATP (0.0001 equivalents or less) are required. In addition to canonical NTPs, the cascade can generate ribose‐ and base‐modified NTP analogues. This is demonstrated through the synthesis of a set of eleven modified nucleotides, including the four 2′‐fluoro‐2′‐dNTPs and nucleotides with 2,6‐diaminopurine as the nucleobase. Production of an essential component for mRNA therapeutics, *N*1‐methyl‐ΨTP (m^1^ΨTP), is demonstrated on a milligram scale.

## Graphical abstract

Nucleoside‐5′‐triphosphates (NTPs) are essential components for the generation of nucleic acids, yet their chemical synthesis is hindered by the low yield of nucleoside phosphorylation. Here, we present a universal, two‐enzyme nucleoside‐to‐NTP cascade that is driven by polyphosphate. This process enables the synthesis of the canonical building blocks of RNA and DNA, as well as ribose‐ and base‐modified NTPs.

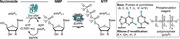

## 1 Introduction

In nature, nucleoside‐5′‐triphosphates (NTPs) are the building blocks used by polymerases to generate nucleic acids. Chemically modified NTPs are the foundation of emerging technologies, including mRNA [1, 2, 3] or siRNA therapeutics [4], aptamers [5, 6, 7], epigenetic sequencing [8, 9] or enzymatic synthesis of modified nucleic acids [10, 11, 12, 13, 14, 15, 16].

**FIGURE 1:**
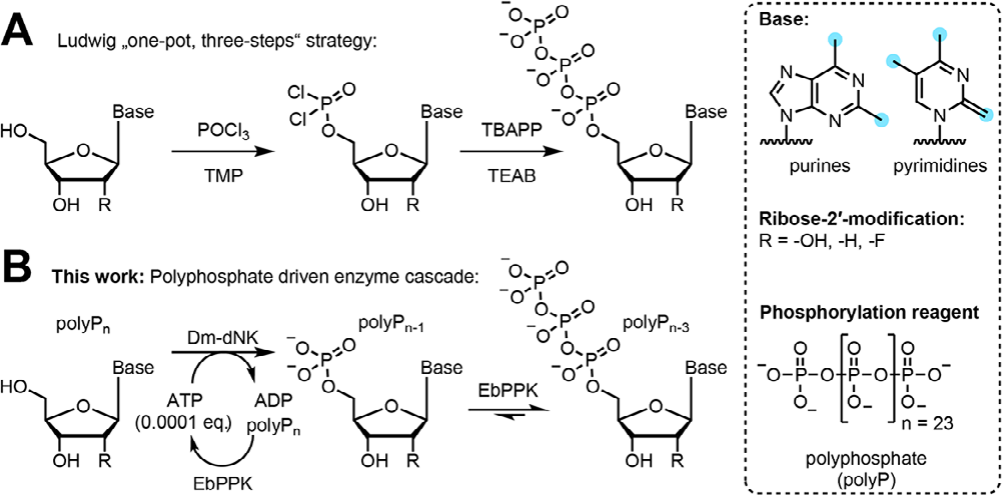
Comparison of chemical and enzymatic strategies for nucleoside‐to‐NTP conversion. (A) Scheme of the chemical synthesis of NTPs using the Ludwig strategy [18]. Phosphorus oxychloride (POCl_3_) in trimethyl phosphate (TMP) is used to prepare the phosphodichloridate intermediate [17], which can be reacted with tributylammonium pyrophosphate (TBAPP) and quenched with triethylammonium bicarbonate (TEAB) buffer [19, 20]. (B) Scheme of the polyphosphate‐driven enzymatic nucleoside‐to‐NTP conversion. The deoxynucleoside kinase from *Drosophila melanogaster* (Dm‐dNK) can convert a broad spectrum of nucleosides to the corresponding NMPs using catalytic amounts of ATP (0.0001 equivalents). The ATP is recycled by the polyphosphate kinase 2 from the *Erysipelotrichaceae* bacterium (EbPPK) using polyphosphate. EbPPK converts a broad spectrum of NMPs to the corresponding NDPs, NTPs and higher phosphorylated species.

The chemical synthesis of modified NTPs is widely used but is hindered by the low yield of nucleoside phosphorylation, which is typically performed as the final synthesis step (Figure 1A) [17, 18, 19, 20]. There has been a recent surge in the use of enzyme cascades for nucleoside‐to‐NTP conversion, as a robust, less hazardous and more environmentally friendly alternative [21, 22, 23]. This approach required three different kinases to catalyse the reaction from nucleoside (N) to nucleoside‐5′‐monophosphate (NMP) and two consecutive phosphorylations to the NTP [21]. Most kinases have evolved to discriminate strictly between substrates containing ribose or deoxyribose moieties, as well as between substrates with purine or pyrimidine nucleobases. With each of the enzymes showing a narrow substrate tolerance, universal solutions have remained scarce [21, 22] or required different enzyme combinations for the production of each NTP [24]. Moreover, deoxynucleoside kinases used for the initial 5′‐monophosphorylation of nucleosides rely on ATP as a co‐factor, which needs to be supplied stoichiometrically or recycled by another kinase, leading to complex mixtures of ATP and NTP analogues that are tedious to separate [21]. The use of costly and instable co‐factors, such as acetyl phosphate, phosphoenolpyruvate or ATP, poses a further challenge to enzymatic approaches [24, 25, 26].

Pioneering work on nucleoside‐to‐NTP conversion from Benčić et al. showed that different nucleoside kinases can be combined with two polyphosphate kinases (PPKs) from classes I and II into three‐enzyme cascades that enabled the generation of canonical rNTPs and base‐modified rNTPs within 24 h [21]. Becker et al. combined a nucleoside kinase with PPKs, which enabled simultaneous generation of ATP and GTP from the corresponding nucleosides [27]. In a recent report, Meng et al. elegantly combined an engineered acid phosphatase (PhoC) with PPKs and an acetate kinase (AcK) in a three‐enzyme system to synthesise modified NTPs on a preparative scale (>100 mg). This approach is ATP‐free, but the kinases require three different co‐factors, namely, pyrophosphate, polyphosphate and acetyl phosphate. The semicontinuous process requires the adjustment of pH, the precipitation of pyrophosphate and the removal of enzymes between the reactions [22].

For the enzymatic nucleoside monophosphorylation, the deoxynucleoside kinase from *Drosophila melanogaster* (Dm‐dNK), first described in 1998, has been shown to have a very broad substrate scope, including all canonical ribo‐ and deoxynucleosides [28]. Due to the high catalytic efficiency of Dm‐dNK in comparison to other deoxynucleoside kinases, even the less‐preferred ribonucleosides (rN) can be converted efficiently. Dm‐dNK has been extensively characterised biochemically and structurally [29, 30, 31, 32, 33].

The PPK enzymes from family 2 class III can perform two consecutive phosphorylation reactions of NMPs to NTPs using polyphosphate [34, 35, 36] and typically approach an equilibrium of ∼8% NMP, ∼22% NDP and ∼70% NTP [26]. By shifting the equilibrium further to the product side, nucleoside‐5′‐tetraphosphates (N4Ps) and higher phosphorylated species are formed, indicating an equilibrium between the chain length of polyP and nucleoside‐5′‐polyphosphate [36, 37, 38]. In contrast to other phosphorylation co‐factors, sodium polyphosphate (polyP) is inexpensive, non‐toxic, bench‐stable and available in bulk. In recent years, many PPKs have been used as efficient ATP recycling systems and for the phosphorylation of modified nucleotides [22, 37, 39, 40, 41, 42]. While some PPK2‐III have been recently reported for the “universal” production of all canonical rNTPs, no PPK2‐III has been reported to efficiently produce all canonical dNTPs [22, 36, 39, 42, 43, 44]. Here, we combine Dm‐dNK with a PPK from an uncharacterised *Erysipelotrichaceae* bacterium (EbPPK) [37, 41, 42, 45]. EbPPK can simultaneously (i) recycle the catalytic amounts of ATP required as a cofactor by Dm‐dNK and (ii) convert NMPs to NDPs and NTPs (Figure 1B).

## 2 Results

**FIGURE 2:**
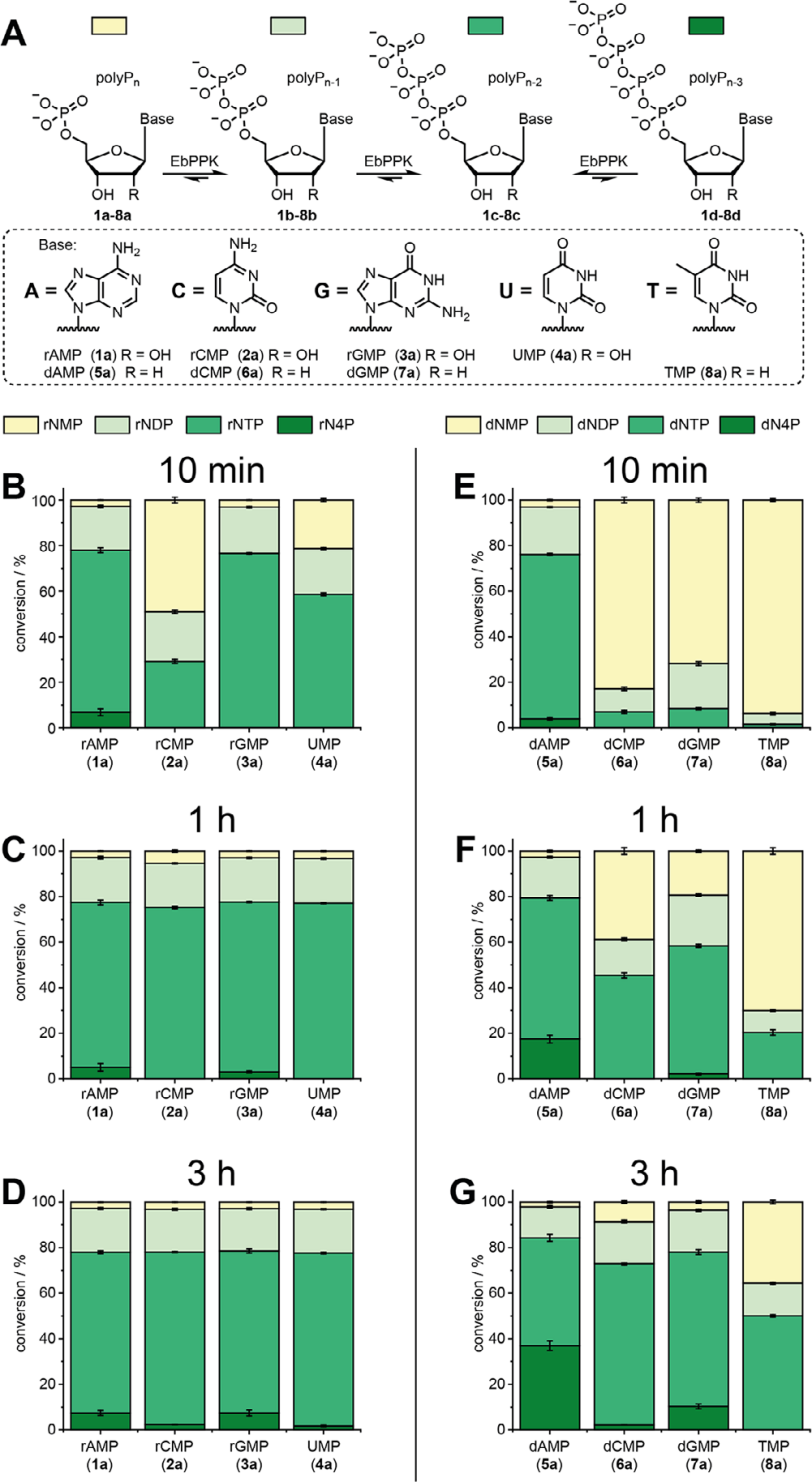
Conversion of the canonical NMPs to the corresponding NDPs, NTPs and N4Ps by EbPPK. (A) Scheme of the EbPPK‐catalysed reaction. (B–D) Conversion of canonical rNMPs by EbPPK at indicated time points. (E–G) Conversion of canonical dNMPs by EbPPK at indicated time points. Conditions: 1 mM of NMPs, 10 µM EbPPK and 4 mg/mL sodium polyphosphate (polyP, Supelco, Cat.‐No. 1.06529, average chain length of 25 phosphate units determined by ^31^P‐NMR, Figure S4). Buffer: 20 mM Tris‐HCl (pH = 8 at 25°C), 20 mM MgCl_2_. Average of three independent replicates with standard deviation (SD).

We approached this challenge in a bioretrosynthetic manner, trying to identify an enzyme capable of synthesising nucleoside‐5′‐triphosphates (NTPs) from the corresponding nucleoside‐5′‐monophosphates (NMPs) first. Starting from 1 mM canonical rNMPs (**1a**‐**4a**) or dNMPs (**5a**‐**8a**), we wondered whether EbPPK (10 µM) was able to convert the substrates to the corresponding NTPs (Figure 2). rNMPs were converted quickly to the corresponding rNDPs and rNTPs (Figure 2B–D). The reaction approaches an equilibrium with conversions of 72% rATP, 75% rCTP, 75% rGTP and 77% UTP after 1 h with only minor changes at 3 h.

To our delight, dNMPs were also converted by EbPPK (Figure 2E–G). At 10 min, a more pronounced trend with production of dATP >> dGTP > dCTP > TTP can be seen. While conversion to dATP (72%) is best at 10 min, dCTP reached 71% at 3 h. Conversions to dGTP (**7c**) reached 68% at 3 h with 10% overreaction to the tetraphosphate dG4P (**7d**). TTP (**8c**) production is slower, with 65% conversion reached only after 24 h (Figure S1). HPLC chromatograms for all analytes are found in Figures S2 and S3, and conversions are summarised in Table S1. As a general trend, EbPPK has a strong preference for rNMPs over their dNMP counterparts. Additionally, NMPs with purine bases are preferred over NMPs with pyrimidine bases.

**FIGURE 3:**
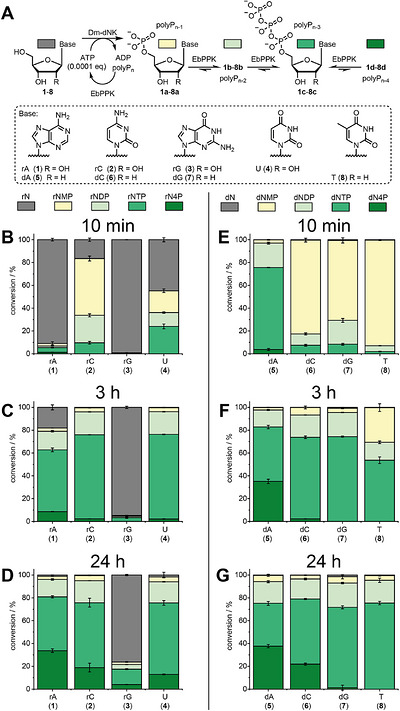
Nucleoside‐to‐NTP cascade reaction using Dm‐dNK/EbPPK. (A) Scheme of the Dm‐dNK/EbPPK cascade reaction. (B–D) Conversion of canonical ribonucleosides (**1**‐**4**) by the Dm‐dNK/EbPPK cascade at indicated time points. (E–G) Conversion of canonical deoxynucleosides (**5**‐**8**) by the Dm‐dNK/EbPPK cascade at indicated time points. Conditions: 1 mM nucleoside, 10 µM Dm‐dNK, 10 µM EbPPK, 0.1 µM ATP, 4 mg/mL polyP. Buffer: 20 mM Tris‐HCl (pH = 8 at 25°C), 20 mM MgCl_2_. Average of three independent replicates with SD.

Previously, we used the Yoshikawa protocol [17, 19] to synthesise NMPs from nucleosides as substrates for EbPPK [37, 41]. Although this reaction results in good yields, it requires laborious preparation of reagents and workup of the reaction with high environmental impact. Therefore, we aimed to find an enzyme that can be combined with EbPPK in a nucleoside‐to‐NTP cascade. The deoxynucleoside kinase from *D. melanogaster* (Dm‐dNK) has been described to have a broad substrate scope, with a preference for pyrimidine over purine nucleosides as well as a preference for deoxynucleosides over ribonucleosides [28]. It is important to emphasise that Dm‐dNK and EbPPK therefore show exactly opposing preferences. Dm‐dNK requires ATP as a cofactor, but we found that the addition of catalytic amounts of ATP (0.1 µM, 0.0001 equivalents) is sufficient if EbPPK is added for polyP‐dependent ATP recycling. The Dm‐dNK/EbPPK cascade enables a polyphosphate‐driven nucleoside‐to‐NTP reaction in one‐pot as single operation (Figure 3A). Starting from the canonical ribonucleosides (**1**‐**4**), conversion to the rNMPs (**1a**‐**4a**) and further reaction to the rNTPs (**1c**‐**4c**) proceed with different rate‐limiting reactions, reflecting the opposing preferences of Dm‐dNK and EbPPK. While purine nucleosides (**1** and **3**) are slowly converted to the rNMPs (**1a** and **3a**), the reaction to the rNTPs (**1c** and **3c**) proceeds faster. The pyrimidine nucleosides (**2** and **4**), on the other hand, are quickly converted to the rNMPs (**2a** and **4a**), but the reaction to the corresponding rNTPs (**2c** and **4c**) is rate‐limiting. (Figure 3B–D).

For rC (**2**) and U (**4**), the remaining ribonucleoside starting material can be observed at the 10 min time point, but efficient conversion to 74% rCTP (**2c**) and 74% UTP (**4c**) are found at 3 h. In the case of rA (**1**), slow conversion to the rAMP (**1a**) limits the conversion to rATP (**1c**) to 54% after 3 h, with 9% overreaction to rA4P (**1d**). No efficient production of rGTP (**3c**) can be observed for the cascade reaction, mainly limited by the inability of Dm‐dNK to produce rGMP (**3a**). Even after 24 h, 76% rG (**3**) remains unchanged in the reaction mixture with only 13% of rGTP (**3c**) and 4% of rG4P (**3d**). For rGTP production, Dm‐dNK should be replaced by nucleoside kinases with a preference for guanosine, such as the nucleoside kinase from *Methanocaldococcus jannaschii* (MjNK) [21, 27, 46].

Starting from deoxynucleosides (**5**‐**8**), the conversion to the corresponding dNMPs by Dm‐dNK is nearly completed after 10 min (Figure 3E–G). Further reaction to the canonical dNTPs (**5c**‐**8c**) is therefore solely dependent on the preference of EbPPK. At 10 min, dA (**5**) is converted to 72% dATP (**5c**), as both Dm‐dNK and EbPPK work efficiently. At 3 h, conversions to 74% dGTP (**7c**) and 72% dCTP (**6c**) are achieved. At 24 h, 75% conversion to TTP is observed. HPLC analysis of cascade reactions can be found in Figures S5 and S6 and Table S2. The identity of the nucleotides formed was confirmed via LC‐TOF‐MS (Figures S7–S14). Although clear substrate preferences for Dm‐dNK and EbPPK can be observed, this cascade is able to efficiently convert all canonical nucleosides containing ribose as well as deoxyribose, except rG (**3**). To the best of our knowledge, this is the first report of a universal polyphosphate‐driven nucleoside‐to‐NTP cascade using only two enzymes that robustly leads to conversions >70% for rCTP, UTP, dATP, dCTP, dGTP and TTP.

**FIGURE 4:**
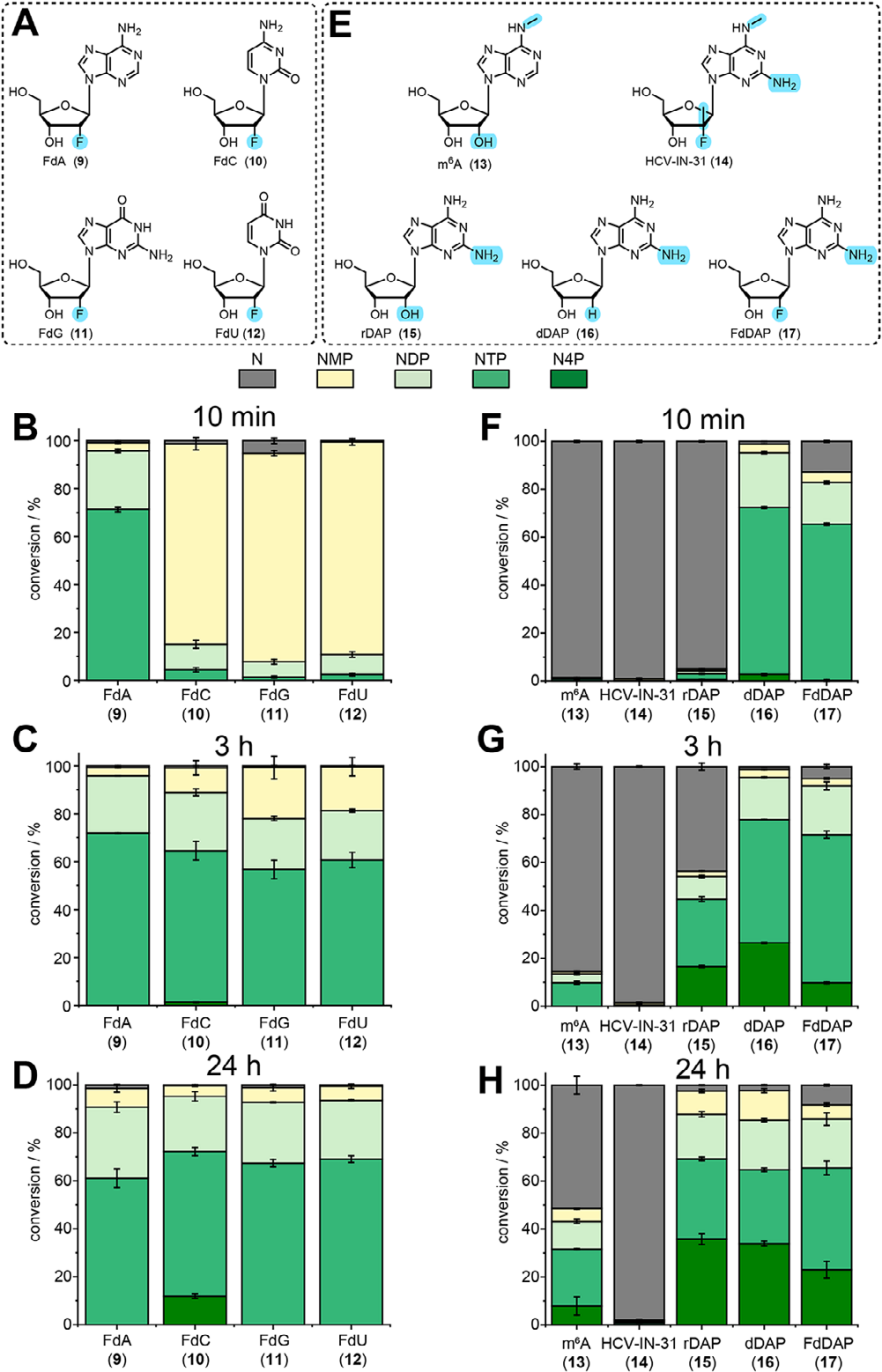
Nucleoside‐to‐NTP cascade for the synthesis of modified NTPs. (A) Panel of 2′‐fluoro‐2′‐deoxynucleosides with canonical nucleobases (**9**‐**12**). (B–D) Conversions of 2′‐fluoro‐2′‐deoxynucleosides at indicated time points. (E) Panel of modified nucleosides with modification of the nucleobase or multiple modifications. (F–H) Conversions of modified nucleosides at indicated time points. Conditions: 1 mM nucleoside (**9**‐**17**), 10 µM Dm‐dNK, 10 µM EbPPK, 0.1 µM ATP, 4 mg/mL polyP. Buffer: 20 mM Tris‐HCl (pH = 8 at 25°C), 20 mM MgCl_2_. Average of three independent replicates with SD.

With the Dm‐dNK/EbPPK cascade in hand, we wondered whether it could be used to produce modified NTPs. As important building blocks for siRNA therapeutics, we aimed to produce the ribose‐modified 2′‐fluoro‐2′‐deoxynucleotides with the canonical nucleobases (**9c**‐**12c**) from the corresponding nucleosides shown in Figure 4A. To our delight, the Dm‐dNK/EbPPK cascade produced all 2′‐fluoro‐2′‐deoxynucleotides (Figure 4B–D). Conversion of FdA (**9**) gave 71% conversion to FdATP (**9c**) within 10 min. After 3 h, the conversion of **10** to FdCTP (**10c**) reaches the maximum of 63%. For FdGTP (**11c**) and FdUTP (**12c**), we find the highest average conversions at 24 h with 67% and 69%, respectively.

Encouraged by these results, we tested a panel of modified nucleosides (Figure 4E–H) to explore the possibilities and limitations of the cascade. First, we tested *N* ^6^‐methyl adenosine (m^6^A, **13**). While m^6^ATP (**13c**) can be produced in 24% conversion, 52% of the nucleoside (**13**) remains unreacted in the reaction mixture after 24 h. Next, we tried to convert the hepatitis C virus inhibitor 31 (HCV‐IN‐31, **14**), a highly modified adenosine derivative. Only trace conversion (0.8%) to the corresponding NTP (**14c**) was found after 24 h, which could be validated by LC‐MS. We turned our attention to nucleosides with 2,6‐diaminopurine (DAP) nucleobase, as these nucleosides are known to be intrinsically fluorescent and enable base pairing with three hydrogen bonds in double‐stranded regions in DNA (DAP:T) and RNA (DAP:U). rDAP (**15**) is slowly converted to 33% rDAPTP (**15c**) and 36% of rDAP4P (**15d**) in 24 h. Luckily, the 2′‐deoxy‐ and 2′‐fluoro‐2′‐deoxynucleosides with DAP base (**16** and **17**) are efficiently converted to the corresponding NTPs. Within 10 min, we observe 70% conversion to **16c,** while 65% conversion to **17c** is found.

These results illustrate that *N* ^6^‐methyl‐modified nucleosides show only modest conversion to the corresponding NTP, while the 2′‐methyl modification nearly abrogates Dm‐dNK activity. The difference in conversion of rDAP (**15**), dDAP (**16**) and FdDAP (**17**) repeatedly shows the preference of Dm‐dNK for 2′‐deoxy‐ and 2′‐fluoro‐2′‐deoxynucleosides over the slower conversion of ribonucleosides. The production of **17c** illustrates that NTPs with dual modification of the nucleobase and the ribose can be efficiently produced, even with wild type enzymes. HPLC analysis is shown in Figures S15–S17 and LC‐TOF‐MS in Figures S18–S26. Conversions are summarised in Table S3.

**FIGURE 5:**
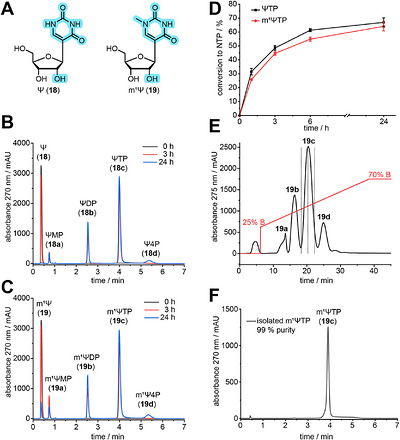
Production of modified NTPs relevant for therapeutic mRNAs. (A) Structure of pseudouridine (Ψ, **18**) and *N*1‐methyl‐pseudouridine (m^1^Ψ, **19**). (B–D) Conversions of Ψ (**18**) and m^1^Ψ (**19**) at indicated time points. (E) Anion exchange chromatograms of the preparative (2 mL, 5 mM nucleoside) reactions for Dm‐dNK/EbPPK cascades starting from m^1^Ψ. Gradient ddH_2_O to 200 mM NaClO_4_ pH = 4.2 (buffer B). Collected fractions containing m^1^ΨTP are indicated with dotted lines. (F) HPLC analysis of the purity of isolated m^1^ΨTP. Conditions: 5 mM pseudouridine (**18**) or *N*1‐methyl‐Ψ (**19**), 10 µM Dm‐dNK, 10 µM EbPPK, 0.1 µM ATP and 4 mg/mL polyP. Buffer: 20 mM Tris‐HCl (pH = 8 at 25°C), 20 mM MgCl_2_.

Next, we aimed to produce NTPs relevant for mRNA therapeutics. The base‐modified nucleosides pseudouridine (Ψ, **18**) and *N*1‐methyl‐Ψ (m^1^Ψ, **19**), shown in Figure 5A, have been shown to reduce the immunogenicity observed with unmodified mRNAs [1, 2, 47]. With a view to future process intensification and scale‐up, we increased the concentration of nucleoside to 5 mM and measured multiple time points to find the optimal conversion to NTPs (Figures 5B–D and S27–S29; Table S4). At 24 h, we find conversions of 67% (ΨTP, **18c**) and 64% (m^1^ΨTP, **19c**).

We performed scale‐up to 2 mL reactions containing 10 µmol of m^1^Ψ (**19**) and separated the reaction mixture over an anion exchange column via low‐pressure chromatography using ÄKTA purifier (Figure 5E). The indicated fractions containing m^1^ΨTP were partially lyophilised, and the product was precipitated with acetone to give **19c** as a white solid (2.3 mg, 4.66 µmol, 47%) in 99% purity (Figures 5F and S30 and S31).

These results illustrate that the enzyme cascade can generate industrially relevant ribose‐modified, base‐modified and dual‐modified NTPs. For m^1^ΨTP, we demonstrate production on a milligram scale with excellent purity. Given the simplicity, robustness and broad substrate scope of our Dm‐dNK/EbPPK cascade, we envision that this approach will become a viable alternative to the established chemical reactions for nucleoside‐to‐NTP conversion.

## 3 Conclusion

We demonstrate that the polyphosphate kinase EbPPK from an uncharacterised *Erysipelotrichaceae* bacterium is a truly universal PPK, accepting all canonical rNMPs and dNMPs. We further developed a strikingly simple two‐enzyme cascade for the universal, polyphosphate‐driven synthesis of canonical and modified nucleoside‐5′‐triphosphates (NTPs). The system combines the promiscuous, ATP‐dependent deoxynucleoside kinase from *D. melanogaster* (Dm‐dNK) with EbPPK. EbPPK fulfils a dual role: it regenerates the catalytic amounts of ATP required by Dm‐dNK (≤ 0.0001 equiv.) and consecutively phosphorylates the resulting (modified) NMPs to the corresponding NTPs.

With this cascade, seven of the eight canonical ribo‐ and deoxynucleosides were efficiently converted to >70% of the corresponding NTPs and N4Ps. Only guanosine, known to be not well accepted by Dm‐dNK [28], gave low conversion to GTP (13%). Importantly, the cascade provides access to modified NTPs. The four 2′‐fluoro‐2′‐deoxy‐NTPs, key building blocks for siRNA therapeutics, aptamers and cyclic dinucleotide analogues, could be efficiently produced. Additionally, we could show that m^6^ATP can be produced. For the antiviral nucleoside, HCV‐IN‐31, only trace amounts (0.8%) of the corresponding NTP were found, illustrating the current limitations of the cascade. A set of nucleosides with 2,6‐diaminopurine (DAP) nucleobase was well converted to the corresponding NTPs. Especially, the DAP derivative with 2′‐fluoro‐2′‐deoxy modification was converted to 65% of the corresponding NTP within only 10 min. This illustrates that the production of NTPs with dual modification of the nucleobase and the ribose can be realised with the cascade. ΨTP and m^1^ΨTP, which are important building blocks for mRNA therapeutics with reduced immunogenicity [1, 2], could be synthesised. For m^1^ΨTP, we perform scale‐up reactions and develop preparative anion exchange chromatography to isolate the compound on a milligram scale in 99% purity. To the best of our knowledge, this constitutes the first nucleoside‐to‐NTP cascade driven solely by polyphosphate that simultaneously accepts ribo‐, deoxy‐, 2′‐fluoro‐2′‐deoxy‐ and base‐modified nucleosides. Of the many different kinases used for nucleoside and nucleotide phosphorylation, only a few have been shown to produce modified NTPs on a preparative milligram scale [21, 22, 25]. In contrast, the chemical synthesis of modified NTPs and N4Ps can be performed on a gram scale [19, 48].

Although Dm‐dNK and EbPPK each exhibit a broad substrate scope, their individual preferences are not perfectly aligned, and ongoing engineering efforts in our laboratory aim to generate variants with matched specificities. In conjunction with recent advances in biocatalytic nucleoside synthesis [49, 50, 51, 52], we anticipate that the present cascade will pave the way towards fully integrated nucleobase‐to‐nucleoside‐to‐NTP routes. Owing to its simplicity, robustness and reliance on a single low‐cost phosphate donor, the Dm‐dNK/EbPPK cascade is poised to accelerate the exploration of the chemical space of modified NTPs and to enable the cost‐efficient production of industrially relevant DNA and RNA building blocks.

## Author Contributions

**Jonathan P. Suess**: data curation, investigation, validation and formal analysis. **Nicolas V. Cornelissen**: conceptualisation, investigation, methodology, validation, formal analysis, supervision, data curation, resources, project administration, visualisation, funding acquisition, writing – original draft, writing – review and editing.

## Conflicts of Interest

The authors declare no conflicts of interest.

## Supporting information



The authors have cited additional references within the Supporting Information [53]. **Supporting File**: anie73018‐sup‐0001‐SuppMat.pdf.

## Data Availability

The data that support the findings of this study are available in the Supporting Information of this article.
