# Supplementary material for: A Universal Nucleoside‐to‐Nucleoside‐5′‐Triphosphate Enzyme Cascade Driven by Polyphosphate
Source: Angew Chem Int Ed Engl. 2026 Jun 11;65(31):e6720227. doi: 10.1002/anie.6720227 (PMC13411211; doi:10.1002/anie.6720227)
Supplement: Supplementary file 1 — The authors have cited additional references within the Supporting Information [53]. Supporting File: anie73018‐sup‐0001‐SuppMat.pdf. [file ANIE-65-e6720227-s001.pdf]

Supporting Information

A Universal Nucleoside-to-Nucleoside-5′-Triphosphate Enzyme Cascade Driven by Polyphosphate

Jonathan P. Suess[a] and Nicolas V. Cornelissen[a,*]

[a]Institute of Biochemistry, University of Münster, Correnstr. 36, D-48149 Münster, Germany.

*E-mail: cornelissen@uni-muenster.de

Table of contents

Materials and methods 3

HPLC anion-exchange method 3

LC-TOF-MS of nucleotides 4

Preparative anion exchange chromatography 5

Polyphosphate kinase 2 from *Erysipelotrichaceae* bacterium (EbPPK) 6

Deoxynucleoside kinase from *Drosophila melanogaster* (Dm-dNK) 7

EbPPK expression and purification 8

Dm-dNK expression and purification 9

EbPPK reactions 10

Dm-dNK/EbPPK cascade reactions 10

Dm-dNK/EbPPK cascade reactions (preparative) 10

Supplementary figures and tables 11

HPLC analysis of EbPPK-catalysed reactions (1a-8a) 12

Table S1: Conversions of EbPPK reactions starting from 1a-8a 14

^{31}P-NMR average chain length determination of sodium polyphosphate 15

HPLC analysis of Dm-dNK/EbPPK cascade reactions (1-8) 16

LC-TOF-MS analysis of Dm-dNK/EbPPK cascade reactions (1-8) 18

Table S2: Conversions of Dm-dNK/EbPPK cascades starting from 1-8. 26

HPLC analysis of Dm-dNK/EbPPK cascade reactions (9-12) 27

HPLC analysis of Dm-dNK/EbPPK cascade reactions (13-17) 28

LC-TOF-MS analysis of Dm-dNK/EbPPK cascade reactions (9-12) 29

LC-TOF-MS analysis of Dm-dNK/EbPPK cascade reactions (13-17) 33

Table S3: Conversions of Dm-dNK/EbPPK cascades starting from 9-17. 38

HPLC analysis of Dm-dNK/EbPPK cascade reactions (18-19) 39

LC-TOF-MS analysis of Dm-dNK/EbPPK cascade reactions (18-19) 40

Table S4: Conversions of Dm-dNK/EbPPK cascades starting from 18-19 (5 mM). 42

Preparative separation of 19a-19d 43

Materials and methods

All chemicals and reagents were purchased from BLD Pharmatech GmbH, Fluorochem, Sigma-Aldrich or VWR and were used without further purification unless otherwise stated. Sodium polyphosphate (polyP, Supelco Cat.-No. 1.06529) was used as the polyphosphate source.

HPLC anion-exchange method

Buffer A	200 mM NaH_2PO_4, pH = 3.0
Buffer B	ddH_2O
Buffer C	Acetonitrile
Flowrate	0.8 mL/min
Temperature	20 °C (column oven)
column	Amaze HA 3x50 mm, 3 µm, 100 Å<br>Helix Chromatography AHA-300531

Min	%A	%B	%C
0	20	50	30
0.5	20	50	30
3	70	0	30
4	70	0	30
4.5	20	50	30
7	20	50	30

Note that with anion-exchange separation, the less anionic analyte elutes first, and the general order of elution is nucleoside, NMP, NDP, NTP and N4P.

LC-TOF-MS of nucleotides

LC-TOF-MS were performed on Bruker maXis II ultra-high resolution QTOF coupled to a Thermo Scientific UltiMate 3000® UHPLC.

Buffer A	50 mM NH_4OAc, pH = 5.7
Buffer B	Acetonitrile
Flowrate	1 mL/min
Temperature	20 °C (column oven)
column	Poroshell 120 EC-C18 column
	3 x 50 mm (1.9 µm)
	Agilent P.N. 699675-302

Min	%A	%B
0	100	0
1	100	0
5	0	100
6.5	0	100
7	100	0
10	100	0

Note that with reverse-phase separation, the most polar analyte elutes first and the general order of elution is N4P, NTP, NDP, NMP and nucleoside.

Preparative anion exchange chromatography

Preparative separations were performed on ÄKTA purifier.

Buffer A	ddH_2O
Buffer B	200 mM Sodium perchlorate pH 4.2
Flowrate	5 mL/min
Temperature	25 °C (room temperature)
column	HiPrep Q FF 16/10 (Cytiva)

Min	%A	%B
0	100	0
2	100	0
injection		
6	100	0
6	75	25
38	30	70
46	30	70

Note that with anion-exchange separation, the less anionic analyte elutes first, and the general order of elution is nucleoside, NMP, NDP, NTP and N4P.

Polyphosphate kinase 2 from *Erysipelotrichaceae* bacterium (EbPPK)

Gene synthesis

pET28a(+)-EbPPK-His$_6$ cloned with NcoI/XhoI restriction sites was ordered at BioCat (Heidelberg) as previously described.[1]

DNA sequence

5'-ATGGCAAATATCTACAAGATCGATAAGCTGAATAACTTTAACCTGAATAACCATAAGACC
GATGATTATAGTCTGTGTAAAGATAAAGACACCGCCCTGGAACTGACCCAGAAAAATATTCAGAAAAT
CTATGACTACCAGCAGAAACTGTATGCCGAAAAGAAAGAAGGTCTGATTATTGCATTTCAGGCAATGG
ATGCAGCCGGCAAAGATGGCACCATTCGCGAAGTGCTGAAAGCACTGGCCCCGCAGGGCGTTCATGAA
AAACCGTTTAAAAGTCCGAGCAGTACCGAACTGGCACATGATTATCTGTGGCGCGTTCATAATGCAGT
GCCGGAAAAAGGTGAAATTACCATTTTTAATCGCAGTCATTACGAAGATGTGCTGATTGGTAAAGTTA
AAGAACTGTATAAGTTCCAGAACAAAGCCGATCGTATTGATGAAAATACCGTTGTGGATAATCGTTAT
GAAGATATTCGTAATTTCGAGAAATACCTGTATAACAATAGCGTTCGCATTATTAAGATCTTTCTGAA
TGTTAGTAAGAAGGAACAGGCAGAACGCTTTCTGAGTCGTATTGAAGAACCGGAAAAGAATTGGAAAT
TTTCAGATAGCGATTTCGAAGAACGTGTTTATTGGGATAAATATCAGCAGGCATTTGAAGATGCCATT
AATGCAACCAGTACCAAAGATTGCCCGTGGTATGTTGTGCCGGCAGATCGCAAATGGTATATGCGTTA
TGTTGTTAGCGAAATTGTGGTTAAAACCCTGGAAGAAATGAATCCGAAATATCCGACCGTTACCAAAG
AAACCCTGGAACGTTTTGAAGGTTATCGTACCAAACTGCTGGAAGAATATAATTATGATCTGGATACC
ATCCGTCCGATTGAAAAACTCGAGCACCACCACCACCACCACTGA-3'

Amino acid sequence

This construct (EbPPK-His$_6$): MW: 36.566 kDa, Molecular extinction coefficient: 57425 $M^{-1} \cdot cm^{-1}$

MANIYKIDKLNNFNLNNHKTDDYSLCKDKDTALELTQKNIQKIYDYQQKLYAEKKEGLIIAFQAMDAA
GKDGTIREVLKALAPQGVHEKPFKSPSSTELAHDYLWRVHNAVPEKGEITIFNRSHYEDVLIGKVKEL
YKFQNKADRIDENTVVDNRYEDIRNFEKYLYNNSVRIIKIFLNVSKKEQAERFLSRIEEPEKNWKFSD
SDFEERVYWDKYQQAFEDAINATSTKDCPWYVVPADRKWYMRYVVSEIVVKTLEEMNPKYPTVTKETL
ERFEGYRTKLLEEYNYDLDTIRPIEKLEHHHHHH

Deoxynucleoside kinase from *Drosophila melanogaster* (Dm-dNK)

The plasmid pET28a(+)-His_6-Dm-dNK was a kind gift from Prof. Jennifer Andexer (University of Freiburg).

DNA sequence

5’–ATGGGCAGCAGCCATCATCATCATCATCACAGCAGCGGCCTGGTGCCGCGCGGCAGCCAT
ATGGCAGAAGCAGCCAGCTGCGCACGCAAAGGCACCAAATATGCCGAAGGTACCCAGCCGTTTACCGT
TCTGATTGAAGGCAATATTGGCAGTGGCAAAACCACCTATCTGAATCATTTTGAAAAGTATAAGAACG
ACATCTGCCTGCTGACCGAACCGGTGGAAAAATGGCGCAATGTTAATGGTGTTAATCTGCTGGAACTG
ATGTATAAAGATCCGAAAAAATGGGCAATGCCGTTTCAGAGTTATGTGACCCTGACCATGCTGCAGAG
CCATACCGCACCGACCAATAAGAAACTGAAAATTATGGAACGTAGCATTTTCAGTGCACGCTATTGCT
TTGTTGAAAATATGCGCCGTAATGGTAGTCTGGAACAGGGCATGTATAATACCCTGGAAGAATGGTAT
AAATTCATTGAAGAAAGCATCCATGTGCAGGCAGATCTGATTATCTATCTGCGTACCAGTCCGGAAGT
TGCATACGAACGTATTCGCCAGCGTGCACGCAGCGAAGAAAGCTGTGTTCCGCTGAAATATCTGCAGG
AACTGCATGAACTGCATGAGGATTGGCTGATTCATCAGCGTCGTCCGCAGAGCTGCAAAGTGCTGGTG
CTGGATGCAGATCTGAATCTGGAAAATATTGGCACCGAATATCAGCGTAGCGAAAGTAGCATTTTTGA
TGCAATTAGTAGCAATCAGCAGCCGAGCCCGGTGCTGGTGAGTCCGAGTAAACGTCAGCGTGTGGCAC
GCTAA–3’

Amino acid sequence

This construct (His_6-Dm-dNK): MW: 31.252 kDa, Molecular extinction coefficient: 40130 $M^{-1}\cdot cm^{-1}$

MGSSHHHHHHSSGLVPRGSHMAEAASCARKGTKYAEGTQPFTVLIEGNIGSGKTTYLNHFEKYKNDIC
LLTEPVEKWRNVNGVNLLELMYKDPKKWAMPFQSYVTLTMLQSHTAPTNKKLKIMERSIFSARYCFVE
NMRRNGSLEQGMYNTLEEWYKFIEESIHVQADLIIYLRTSPEVAYERIRQRARSEESCVPLKYLQELH
ELHEDWLIHQRRPQSCKVLVLDADLNLENIGTEYQRSESSIFDAISSNQQPSPVLVSPSKRQRVAR

EbPPK expression and purification

EbPPK was expressed and purified as previously described and yielded 125 mg of soluble protein per litre of culture.[1-2]

In short:

For EbPPK production, *E. coli* BL21(DE3) cells were transformed with either pET28a(+)-EbPPK-His_6 by electroporation, regenerated in SOC media for 1 h at 37 °C and 300 rpm, cultivated in 200 mL in LB medium with kanamycin (50 µg/mL) at 37 °C and 180 rpm for 16 h. 200 mL overnight culture were added to 4 L of LB medium with kanamycin and grown to an OD_{600} = 0.8. Expression was induced with 0.5 mM IPTG at 20 °C for 16 h. Cells were harvested by centrifugation (5000 xg, 4 °C, 30 min) and pellets were stored at −70 °C.

Pellets were resuspended in Buffer A (50 mM Tris-HCl, pH 7.5, 300 mM NaCl, 10 mM imidazole, 10 % glycerol, filtered, degassed) and lysed by sonication (40 % amplitude, 0.5 second on/off pulses for 3 min, 3 times) by a Sonopuls GM3100 (Bandelin). Cell debris was removed by centrifugation (11000 xg, 4 °C, 30 min) and the supernatant was filtered through a 0.2 µm syringe filter and loaded into a Superloop™. Protein purification was performed by IMAC using a HisTrap FF 5 mL (GE Healthcare) column connected to an ÄKTA Purifier system (GE Healthcare). The protein was eluted with Buffer B (50 mM Tris pH 7.5, 300 mM NaCl, 500 mM imidazole, 10 % glycerol). Fractions containing EbPPK were concentrated, and the buffer was exchanged to Buffer C (50 mM Tris pH 7.5, 300 mM NaCl, 10 % glycerol) using Amicon Ultra-15 centrifuge filters (UFC903024, 30 kDa cut off). The concentration was determined by absorbance at 280 nm (Molecular extinction coefficient: 57425 $M^{-1}\cdot cm^{-1}$). Enzyme solution was diluted to 1 mM (Buffer C), the concentration was confirmed by another absorbance measurement, aliquots were flash frozen in liquid nitrogen and stored at −70 °C.

Dm-dNK expression and purification

For Dm-dNK production, *E. coli* BL21(DE3) cells were transformed with pET28a(+)-His_6-Dm-dNK by electroporation, regenerated in SOC media for 1 h at 37 °C and 300 rpm, cultivated in 200 mL in LB medium with kanamycin (50 µg/mL) at 37 °C and 180 rpm for 16 h. 200 mL overnight culture were added to 4 L of LB medium with kanamycin and grown to an OD_{600} = 0.8. Expression was induced with 0.5 mM IPTG at 20 °C for 16 h. Cells were harvested by centrifugation (5000 xg, 4 °C, 30 min) and pellets were stored at −70 °C.

Pellets were resuspended in Buffer A (50 mM Tris-HCl, pH 7.5, 300 mM NaCl, 10 mM imidazole, 10 % glycerol, filtered, degassed) and lysed by sonication (30 % amplitude, 0.5 second on/off pulses for 3 min, 3 times) by a Sonopuls GM3100 (Bandelin). Cell debris was removed by centrifugation (11000 xg, 4 °C, 30 min) and the supernatant was filtered through a 0.2 µm syringe filter and loaded into a Superloop™. Protein purification was performed by IMAC using a HisTrap FF 5 mL (GE Healthcare) column connected to a ÄKTA Purifier system (GE Healthcare). The protein was eluted with Buffer B (50 mM Tris pH 7.5, 300 mM NaCl, 500 mM imidazole, 10 % glycerol). Fractions containing Dm-dNK were concentrated, and the buffer was exchanged to Buffer C (50 mM Tris pH 7.5, 300 mM NaCl, 10 % glycerol) using Amicon Ultra-15 centrifuge filters (UFC901024, 10 kDa cut off). The concentration was determined by absorbance at 280 nm (Molecular extinction coefficient: 40130 $M^{-1} \cdot cm^{-1}$). Enzyme solution was diluted to 1 mM (Buffer C), the concentration was confirmed by another absorbance measurement, aliquots were flash frozen in liquid nitrogen and stored at −70 °C.

EbPPK reactions

For EbPPK reactions, 1 mM NMP (**1a-8a**) was incubated in 20 mM Tris (pH 8) with 20 mM $MgCl_2$, 4 g/L polyphosphate and 10 µM EbPPK in a total volume of 50 µL at 30 °C. Samples were taken after indicated timepoints, the enzyme was denatured at 85 °C for 1.5 min, and the samples were centrifuged (21000 xg, 4 °C, 10 min) to remove precipitated protein. The supernatant was analysed *via* HPLC (2 µL injection).

Dm-dNK/EbPPK cascade reactions

For the Dm-dNK/EbPPK cascade reactions, 1 mM nucleoside (**1**-**17**) or 5 mM in the case of **18** and **19** was incubated in 20 mM Tris (pH 8) with 20 mM $MgCl_2$, 4 g/L polyphosphate, 0.1 µM rATP, 10 µM Dm-dNK and 10 µM EbPPK in a total volume of 40 µL at 30 °C. Samples were taken after indicated timepoints, the enzyme was denatured at 85 °C for 1.5 min, and the samples were centrifuged (21000 xg, 4 °C, 10 min) to remove precipitated protein. The supernatant was analysed *via* HPLC (2 µL injection). Note that nucleosides were prepared as 4 mM stock solutions in ddH_2O except for rG (**3**) and dG (**7**) which contained ddH_2O with 4 % DMSO for solubility resulting in 1 % DMSO in the reaction mixture.

Dm-dNK/EbPPK cascade reactions (preparative)

5 mM $m^1\Psi$ (**19**) was incubated in 20 mM Tris (pH 8) with 20 mM $MgCl_2$, 4 g/L polyphosphate, 0.1 µM rATP, 10 µM Dm-dNK and 10 µM EbPPK in a total volume of 2-4 mL at 30 °C for 24 h. The enzyme was denatured at 85 °C for 1.5 min, and the samples were centrifuged (21000 xg, 4 °C, 10 min) to remove precipitated protein. 2-4 mL of the reaction mixture was diluted in ddH_2O to a total volume of 10 mL and loaded into the ÄKTA Superloop™ (10 mL) for preparative separation.

Fractions containing $m^1\Psi$TP (**19c**) were pooled and lyophilised to reduce the volume to ~2 mL. The concentrated solution was split into 1 mL fractions and transferred to 50 mL falcon tubes. $m^1\Psi$TP (**19c**) was precipitated by addition of 50 mL acetone (−20 °C) followed by incubation at −20 °C for 30 min. The NTP was collected by centrifugation (4000 xg, 4 °C, 30 min). The acetone was removed and the white solid was dissolved in 200 µL ddH_2O. The concentration (yield) was determined by

absorbance. The reactions gave m[1]ΨTP (**19c**) as white solid 2.3 mg, 4.67 µmol, 47 % (2 mL reaction) and 3.7 mg, 7.35 µmol, 37 % (4 mL reaction) with 99 % purity.

Supplementary figures and tables

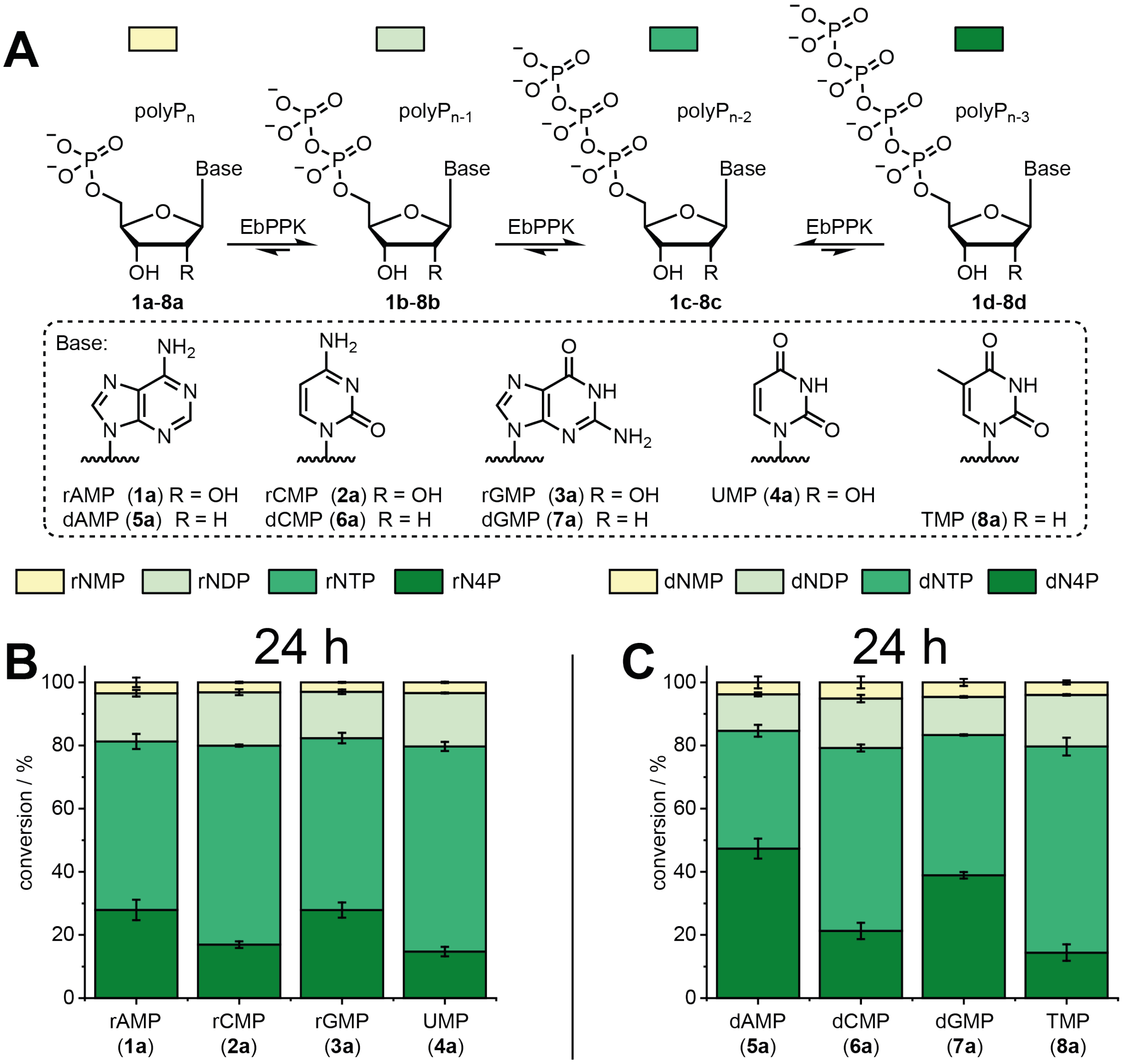

Fig. S1: Conversion of the canonical NMPs to the corresponding NDPs, NTPs and N4Ps by EbPPK. A) Scheme of the EbPPK-catalysed reaction. B) Conversion of canonical rNMPs by EbPPK at 24 h. C) Conversion of canonical dNMPs by EbPPK at 24 h. Additional information to Figure 2 in the main text.

HPLC analysis of EbPPK-catalysed reactions (1a-8a)

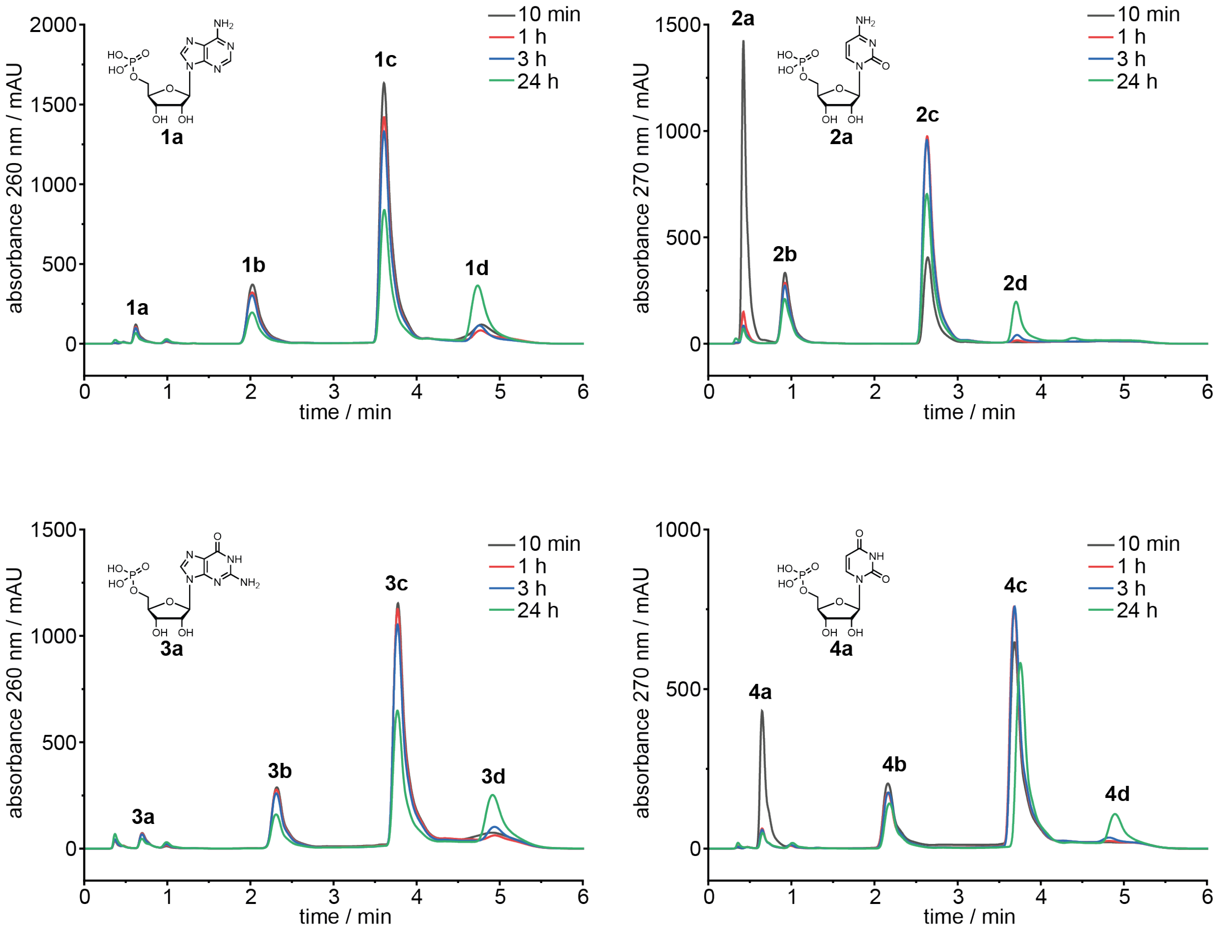

Fig. S2: Representative HPLC analysis of the reaction of EbPPK starting from rNMPs (**1a**-**4a**).

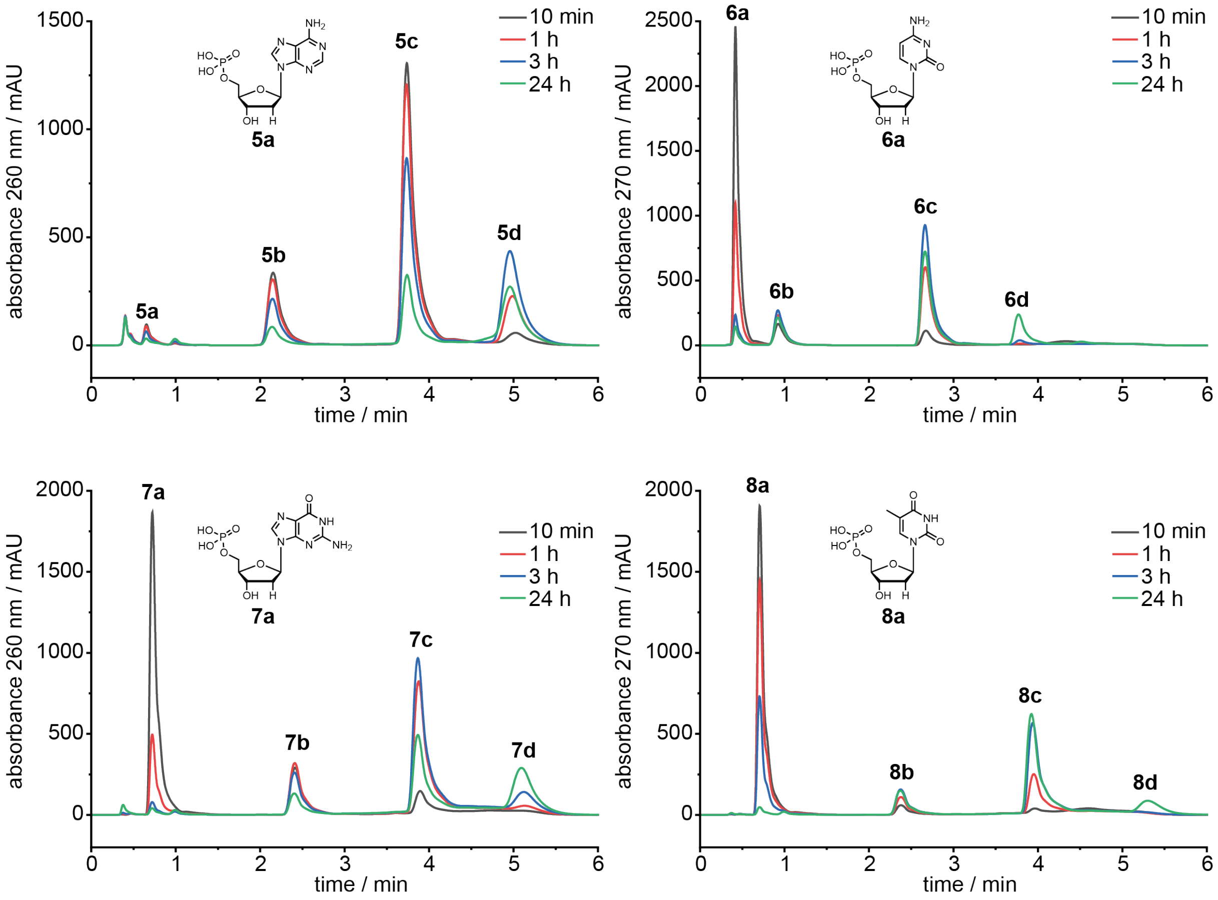

Fig. S3: Representative HPLC analysis of the reaction of EbPPK starting from dNMPs (**5a**-**8a**).

Table S1: Conversions of EbPPK reactions starting from 1a-8a. Average conversion and standard deviation of three independent experiments.

	**a** (NMP)		**b** (NDP)		**c** (NTP)		**d** (N4P)	
**10 min**	conv. (%)	± SD	conv. (%)	± SD	conv. (%)	± SD	conv. (%)	± SD
**1a**	2.8	0.1	19.2	0.6	71.2	1.1	6.8	1.5
**2a**	49.0	1.3	21.8	0.8	29.2	0.8	0.0	0.0
**3a**	3.1	0.1	20.3	0.4	76.6	0.3	0.0	0.0
**4a**	21.3	0.7	20.1	0.4	58.6	0.7	0.0	0.0
**5a**	3.1	0.3	20.8	0.2	72.2	0.5	3.9	0.4
**6a**	82.9	1.2	10.1	0.8	7.0	0.7	0.0	0.0
**7a**	71.7	1.0	19.8	0.9	8.4	0.4	0.0	0.0
**8a**	93.7	0.6	4.7	0.4	1.6	0.2	0.0	0.0

	**a** (NMP)		**b** (NDP)		**c** (NTP)		**d** (N4P)	
**1 h**	conv. (%)	± SD	conv. (%)	± SD	conv. (%)	± SD	conv. (%)	± SD
**1a**	2.8	0.1	19.7	0.6	72.4	1.0	5.0	1.6
**2a**	5.3	0.4	19.4	0.2	75.2	0.6	0.0	0.0
**3a**	3.0	0.1	19.4	0.3	74.5	0.3	3.1	0.5
**4a**	3.3	0.2	19.7	0.4	77.1	0.2	0.0	0.0
**5a**	2.7	0.4	17.9	0.4	62.0	1.1	17.4	1.7
**6a**	38.8	1.5	15.8	0.7	45.5	1.1	0.0	0.0
**7a**	19.3	0.4	22.3	0.5	56.2	0.7	2.2	0.4
**8a**	70.1	1.5	9.6	0.3	20.3	1.2	0.0	0.0

	**a** (NMP)		**b** (NDP)		**c** (NTP)		**d** (N4P)	
**3 h**	conv. (%)	± SD	conv. (%)	± SD	conv. (%)	± SD	conv. (%)	± SD
**1a**	2.8	0.1	19.2	0.5	70.5	0.6	7.4	1.1
**2a**	3.2	0.1	18.8	0.4	75.6	0.3	2.4	0.1
**3a**	2.9	0.1	18.6	0.5	71.1	0.9	7.4	1.3
**4a**	3.1	0.1	19.3	0.2	75.9	0.4	1.7	0.3
**5a**	2.1	0.3	13.6	0.5	47.3	1.5	37.0	2.1
**6a**	8.6	0.5	18.5	0.6	70.7	0.5	2.2	0.0
**7a**	3.6	0.5	18.4	0.5	67.7	1.0	10.3	1.1
**8a**	35.6	0.8	14.3	0.4	50.1	0.5	0.0	0.0

	**a** (NMP)		**b** (NDP)		**c** (NTP)		**d** (N4P)	
**24 h**	conv. (%)	± SD	conv. (%)	± SD	conv. (%)	± SD	conv. (%)	± SD
**1a**	3.5	1.5	15.3	1.1	53.3	2.4	27.9	3.2
**2a**	3.1	0.2	16.9	0.9	63.0	0.3	16.9	1.1
**3a**	3.0	0.2	14.7	0.7	54.5	1.7	27.9	2.4
**4a**	3.4	0.2	17.0	0.1	64.9	1.5	14.7	1.5
**5a**	3.8	1.9	11.5	0.6	37.3	1.9	47.4	3.2
**6a**	5.1	1.9	15.7	1.2	57.9	1.1	21.3	2.6
**7a**	4.6	1.1	12.1	0.2	44.4	0.2	38.9	1.1
**8a**	4.0	0.6	16.4	0.2	65.2	2.8	14.4	2.6

^{31}P-NMR average chain length determination of sodium polyphosphate

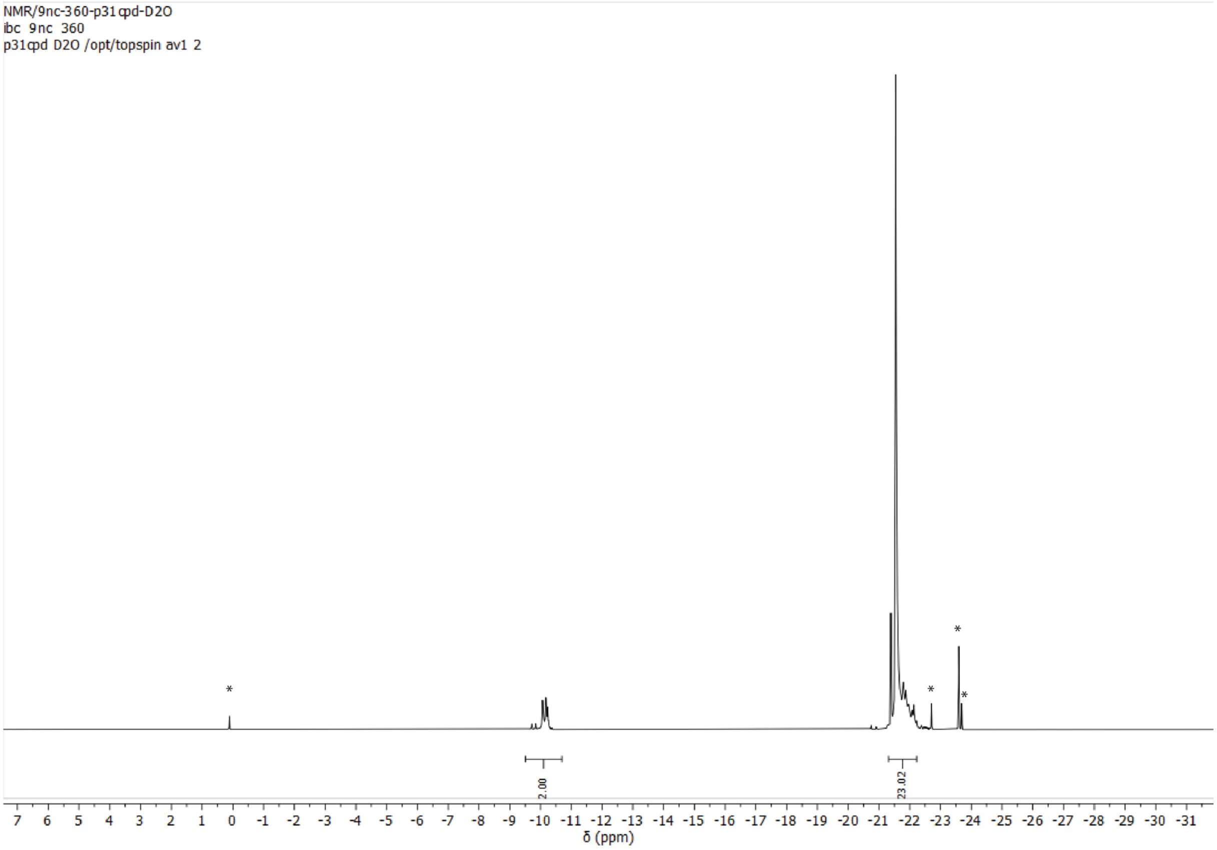

Fig. S4: ^{31}P-NMR spectrum of sodium polyphosphate used (Supelco Product Number 1.06529). Given the heterogeneity of the sodium polyphosphate (polyP, Graham´s salt) observed in the NMR spectrum, we used the method of Christ *et al.*[3] to determine the average chain length. Terminal phosphate groups give rise to signals around −10 ppm, while internal phosphates are seen around −22 ppm. Starred peaks represent small amounts of orthophosphate (left) and cyclic phosphates (right). Setting the integral at −10 ppm to 2 (for the two termini per chain) gives an integration of 23 for the internal phosphates, indicating an average chain length of 25 overall.

Note that many products sold as Graham´s salt or sodium hexametaphosphate are mostly linear polyphosphate with different chain lengths.

HPLC analysis of Dm-dNK/EbPPK cascade reactions (1-8)

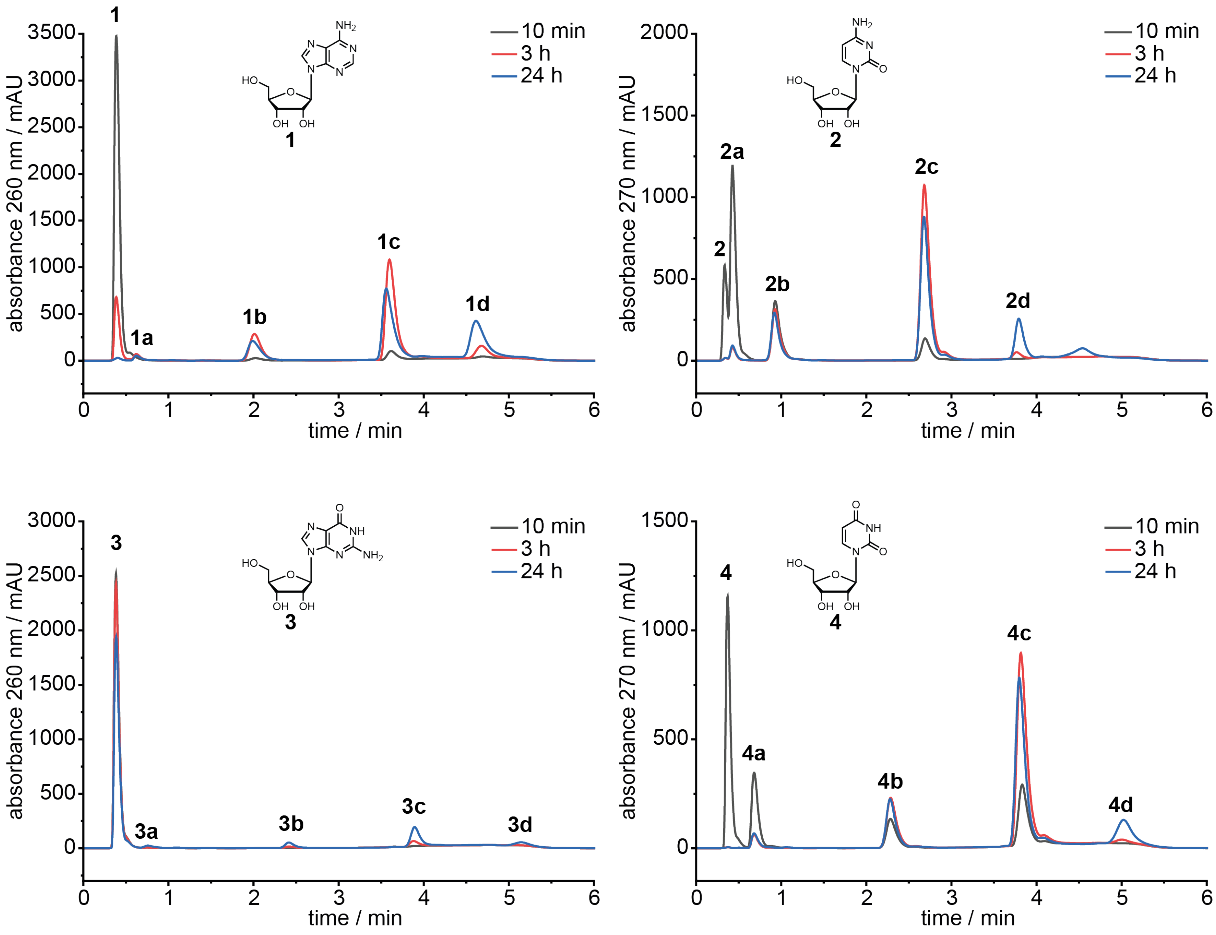

Fig. S5: Representative HPLC analysis of the Dm-dNK/EbPPK cascade reaction starting from ribonucleosides (**1**-**4**).

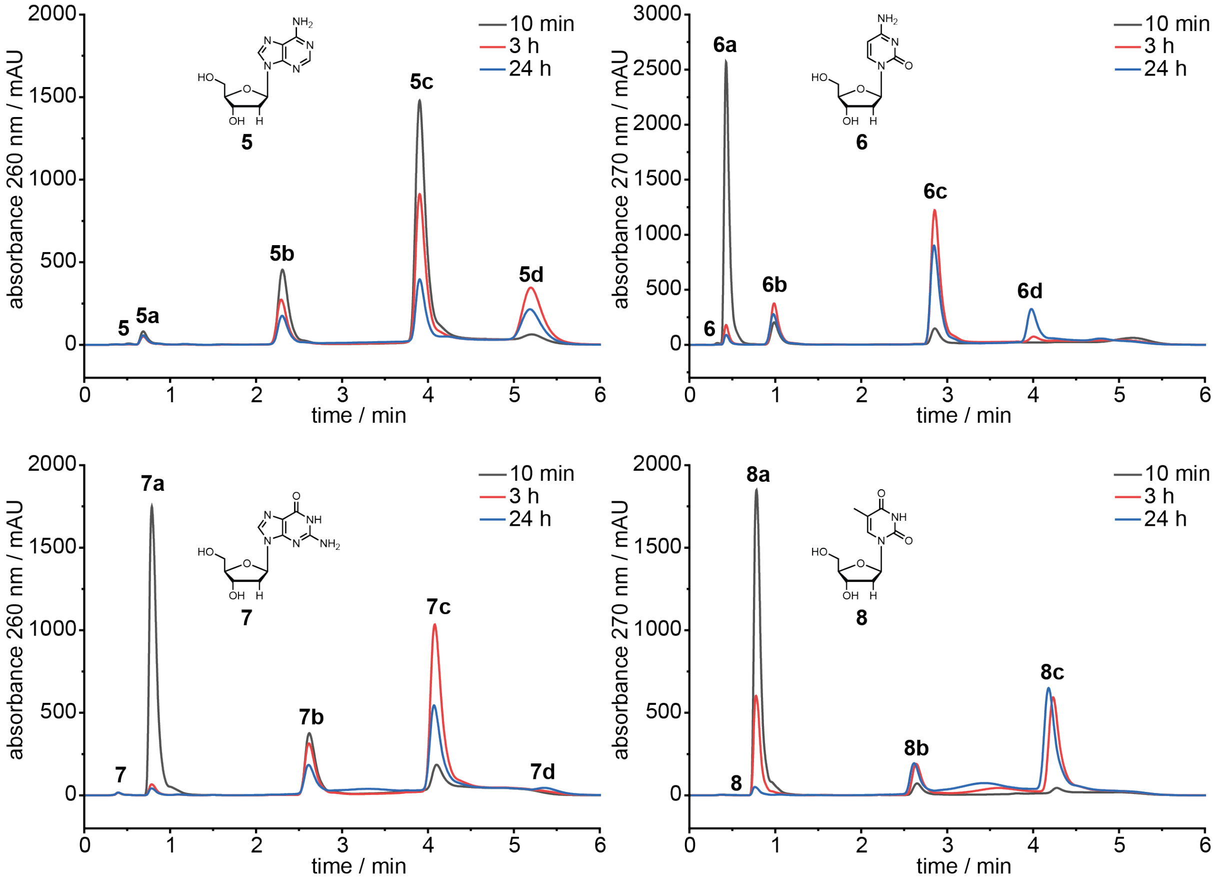

Fig. S6: Representative HPLC analysis of the Dm-dNK/EbPPK cascade reaction starting from deoxyribonucleosides (**5**-**8**).

LC-TOF-MS analysis of Dm-dNK/EbPPK cascade reactions (1-8)

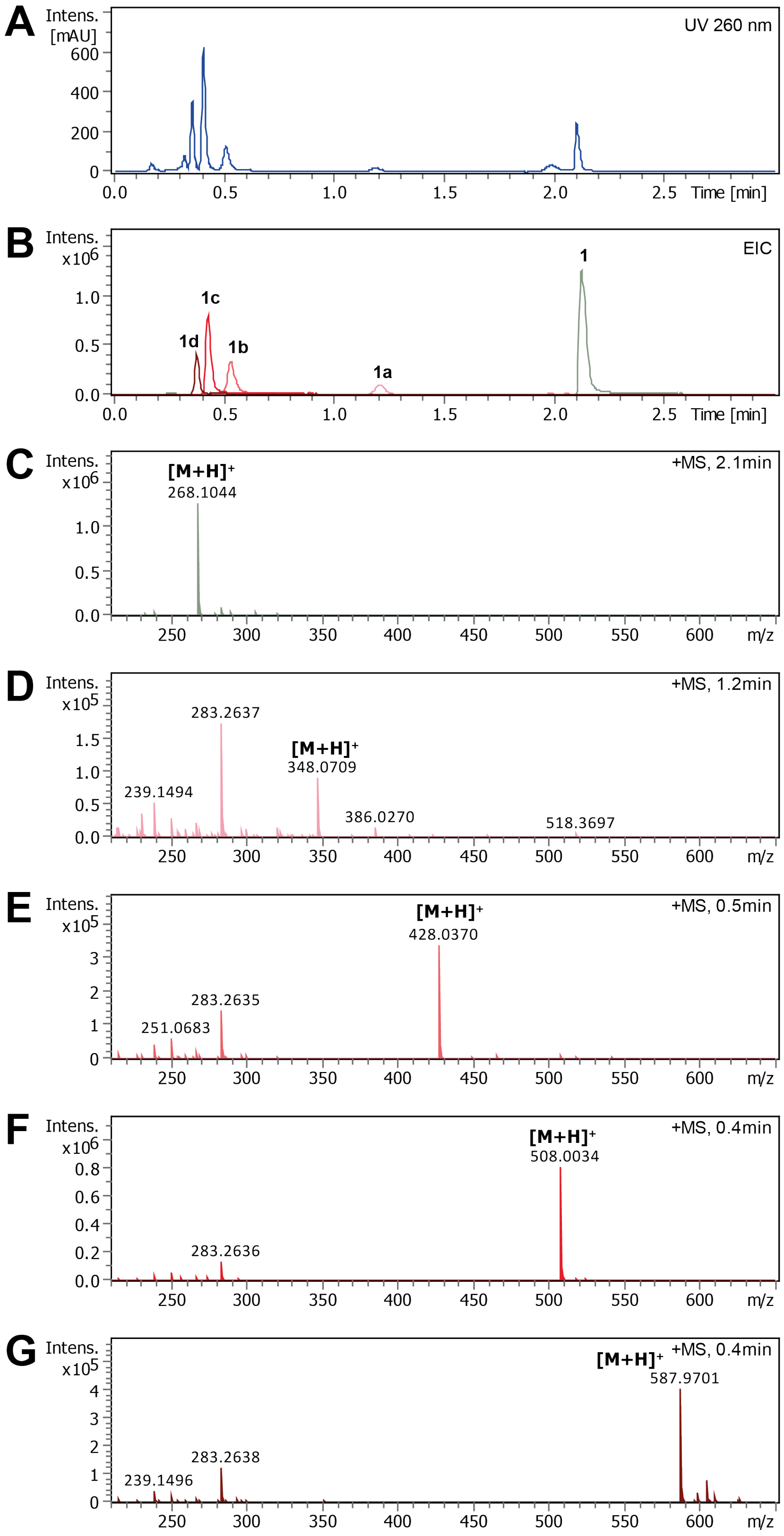

Fig. S7: LC-TOF-MS analysis of the Dm-dNK/EbPPK cascade reaction starting from **1**. A) UV chromatogram at 260 nm. B) Extracted-ion chromatogram for **1-1d**. C)-G) Mass spectrum for **1-1d**.

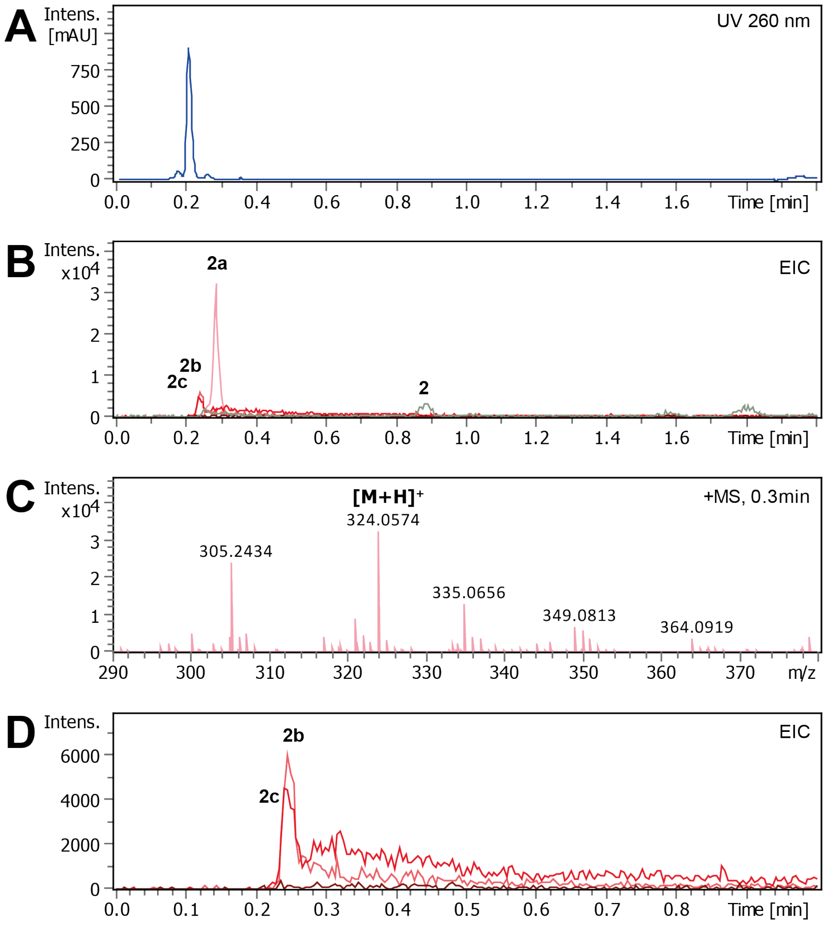

Fig. S8: LC-TOF-MS analysis of the Dm-dNK/EbPPK cascade reaction starting from **2**. A) UV chromatogram at 260 nm. B) Extracted-ion chromatogram for **2**-**2c**. C) Mass spectrum for **2a**. D) Extracted-ion chromatogram for **2b**-**2c**.

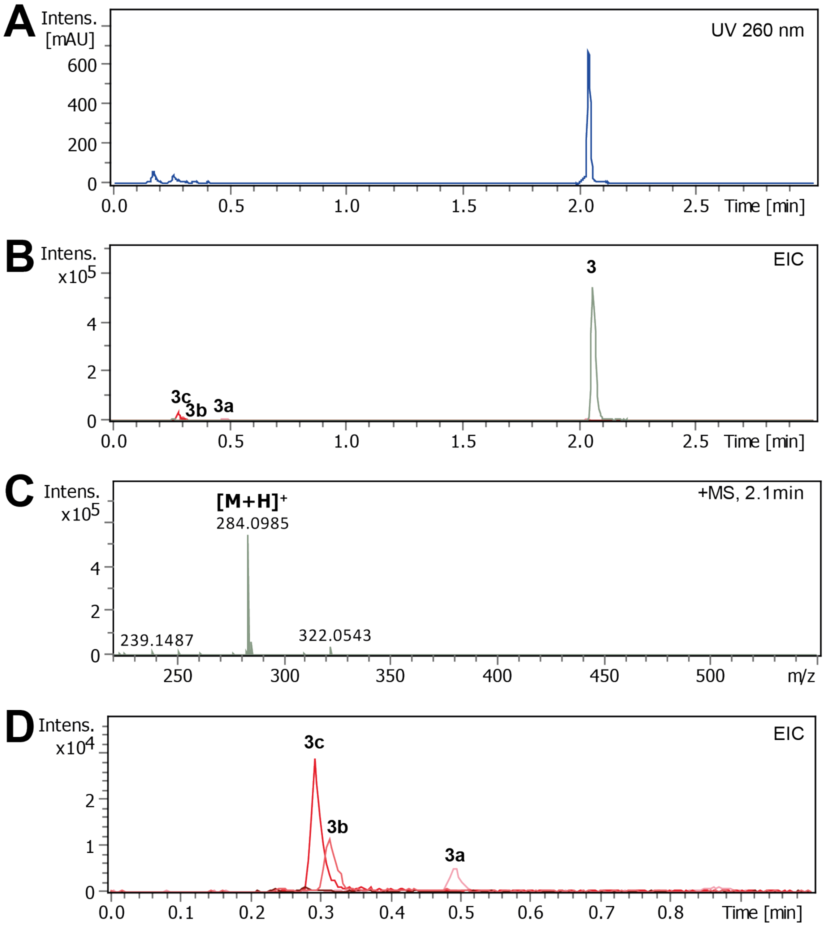

Fig. S9: LC-TOF-MS analysis of the Dm-dNK/EbPPK cascade reaction starting from **3**. A) UV chromatogram at 260 nm. B) Extracted-ion chromatogram for **3**-**3c**. C) Mass spectrum for **3**. D) Extracted-ion chromatogram for **3a**-**3c**.

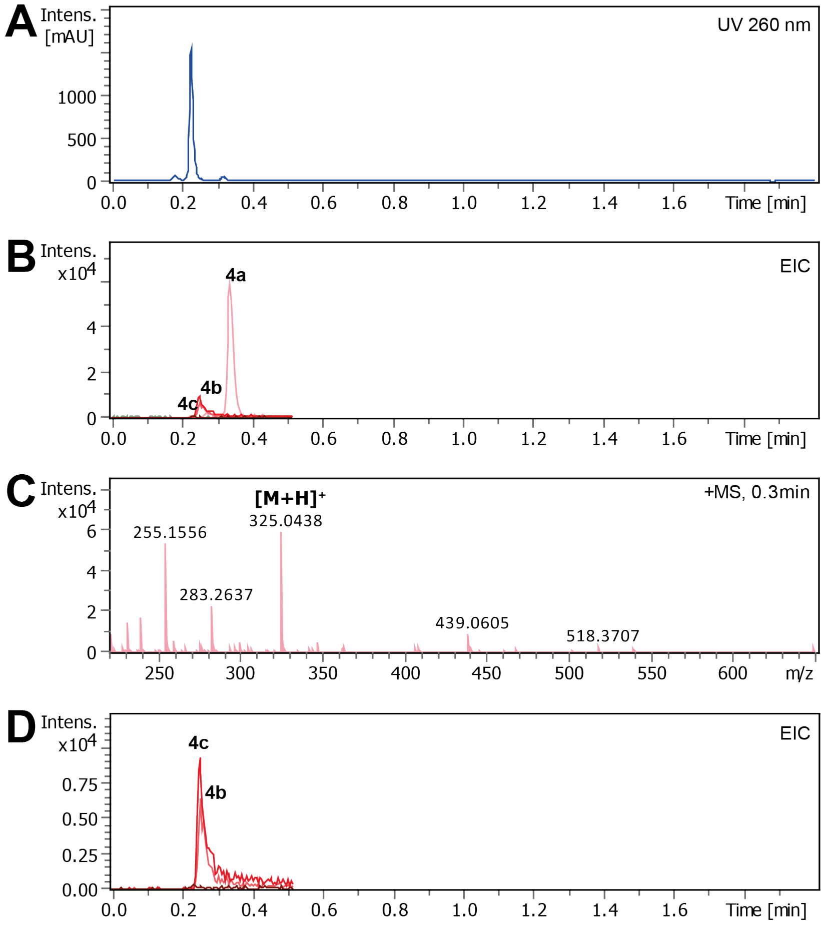

Fig. S10: LC-TOF-MS analysis of the Dm-dNK/EbPPK cascade reaction starting from **4**. A) UV chromatogram at 260 nm. B) Extracted-ion chromatogram for **4a**-**4c**. C) Mass spectrum for **4a**. D) Extracted-ion chromatogram for **4b**-**4c**.

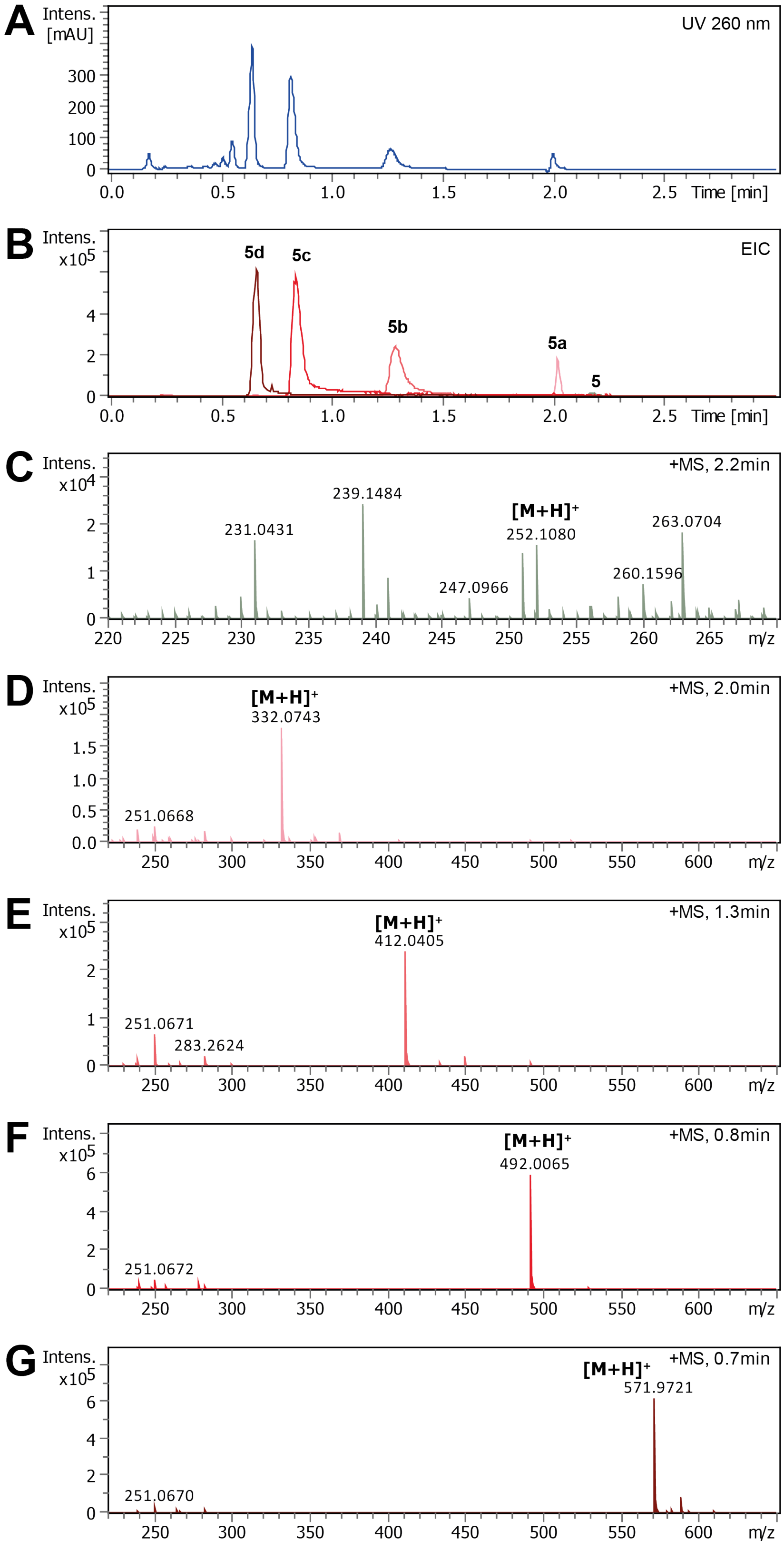

Fig. S11: LC-TOF-MS analysis of the Dm-dNK/EbPPK cascade reaction starting from **5**. A) UV chromatogram at 260 nm. B) Extracted-ion chromatogram for **5**-**5d**. C)-G) Mass spectrum for **5**-**5d**.

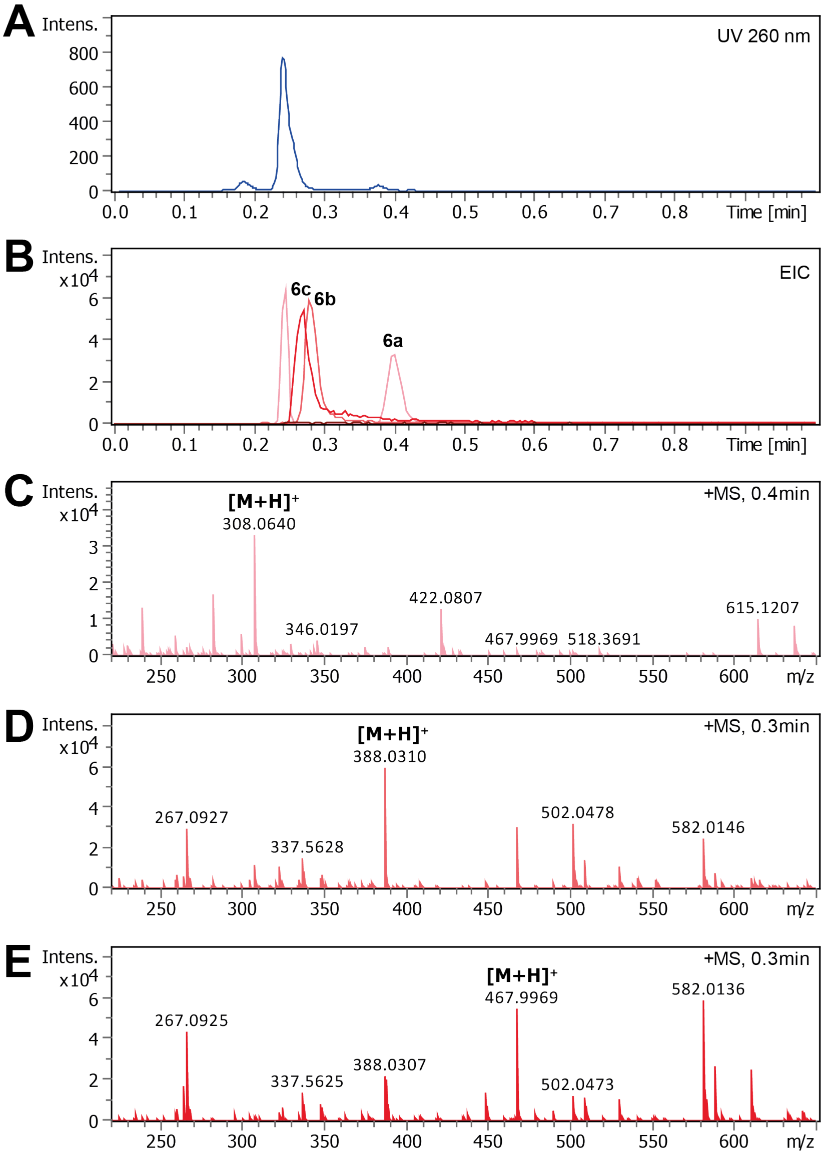

Fig. S12: LC-TOF-MS analysis of the Dm-dNK/EbPPK cascade reaction starting from **6**. A) UV chromatogram at 260 nm. B) Extracted-ion chromatogram for **6a**-**6c**. C)-E) Mass spectrum for **6a**-**6c**.

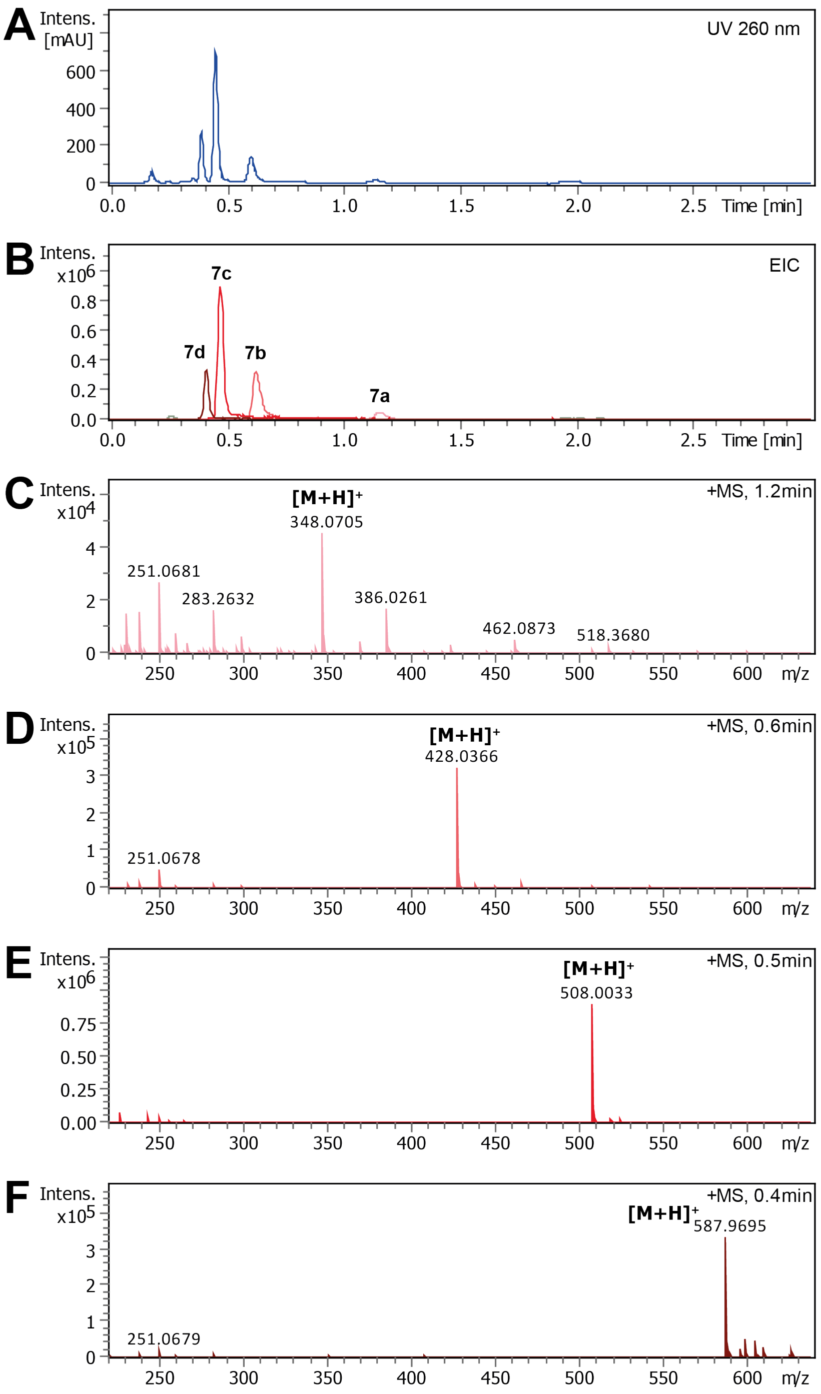

Fig. S13: LC-TOF-MS analysis of the Dm-dNK/EbPPK cascade reaction starting from **7**. A) UV chromatogram at 260 nm. B) Extracted-ion chromatogram for **7a**-**7d**. C)-F) Mass spectrum for **7a**-**7d**.

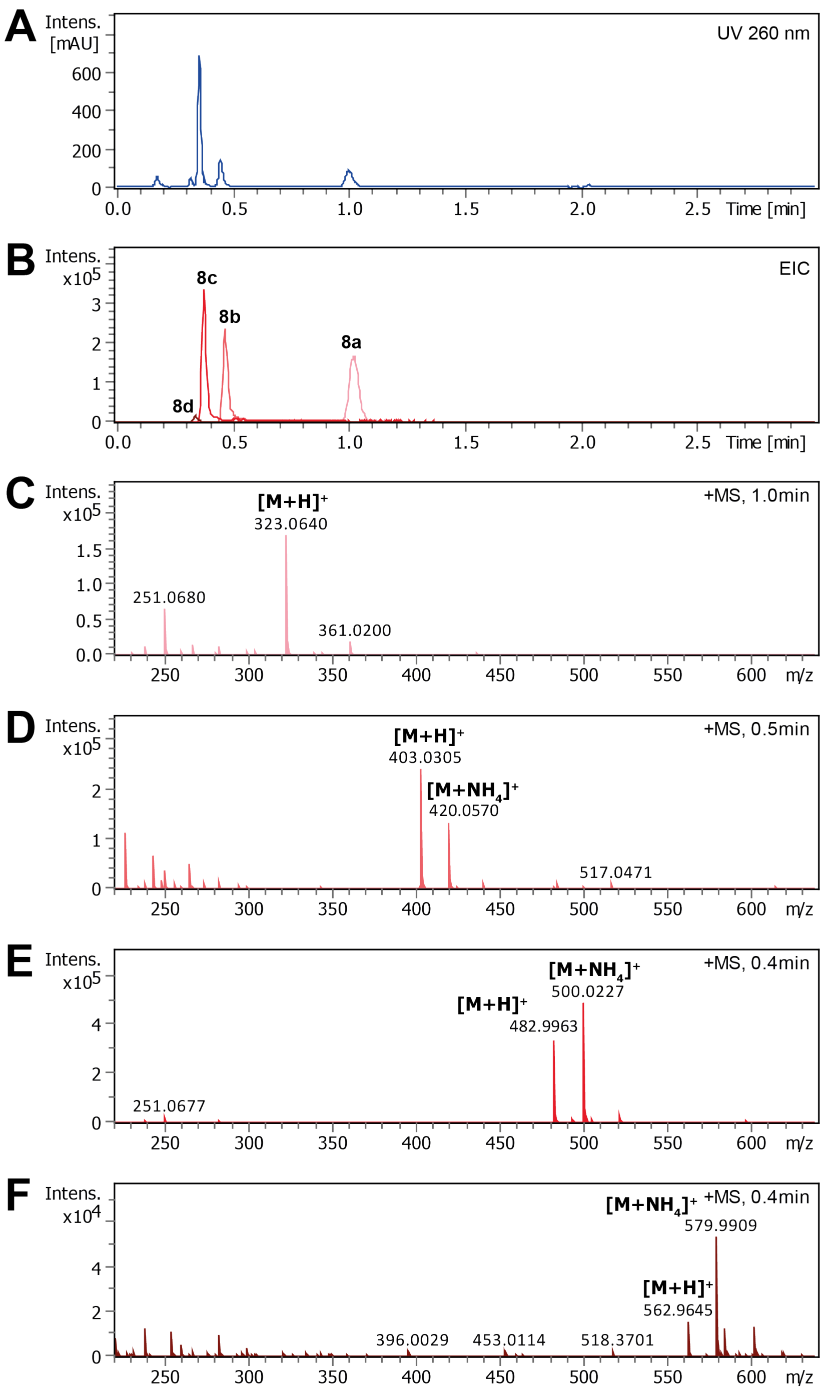

Fig. S14: LC-TOF-MS analysis of the Dm-dNK/EbPPK cascade reaction starting from **8**. A) UV chromatogram at 260 nm. B) Extracted-ion chromatogram for **8a-8d**. C)-F) Mass spectrum for **8a-8d**.

Table S2: Conversions of Dm-dNK/EbPPK cascades starting from 1-8. Average conversion and standard deviation of three independent experiments.

	nucleoside		**a** (NMP)		**b** (NDP)		**c** (NTP)		**d** (N4P)	
**10 min**	conv. (%)	± SD	conv. (%)	± SD	conv. (%)	± SD	conv. (%)	± SD	conv. (%)	± SD
**1**	90.9	1.2	2.2	1.4	1.5	0.1	4.1	0.3	1.3	0.1
**2**	16.5	1.4	49.8	2.1	24.1	1.3	9.7	1.2	0.0	0.0
**3**	99.1	0.1	0.4	0.1	0.2	0.0	0.3	0.0	0.0	0.0
**4**	44.7	1.8	19.1	1.5	12.1	0.6	24.0	2.1	0.0	0.0
**5**	0.1	0.0	2.9	0.3	21.4	0.8	71.8	0.2	3.8	0.9
**6**	0.3	0.0	82.2	1.6	9.9	0.9	7.5	0.8	0.0	0.0
**7**	0.6	0.0	70.1	2.3	20.9	1.7	8.4	0.8	0.0	0.0
**8**	0.3	0.1	92.6	0.3	5.1	0.1	2.0	0.3	0.0	0.0

	nucleoside		**a** (NMP)		**b** (NDP)		**c** (NTP)		**d** (N4P)	
**3 h**	conv. (%)	± SD	conv. (%)	± SD	conv. (%)	± SD	conv. (%)	± SD	conv. (%)	± SD
**1**	18.1	2.2	2.8	0.1	16.3	0.6	54.1	1.6	8.7	0.2
**2**	0.5	0.0	3.4	0.1	20.1	0.2	73.7	0.1	2.3	0.1
**3**	94.8	0.9	0.7	0.1	1.1	0.1	3.4	0.7	0.0	0.0
**4**	0.3	0.1	3.5	0.0	19.9	0.2	74.2	0.2	2.2	0.3
**5**	0.1	0.0	2.2	0.2	14.9	0.4	47.7	1.5	35.1	1.9
**6**	0.1	0.0	6.6	1.3	19.5	0.1	71.5	1.1	2.3	0.1
**7**	0.7	0.0	3.8	0.5	21.1	0.2	74.4	0.4	0.0	0.0
**8**	0.2	0.0	30.4	3.4	15.7	1.1	53.7	2.8	0.0	0.0

	nucleoside		**a** (NMP)		**b** (NDP)		**c** (NTP)		**d** (N4P)	
**24 h**	conv. (%)	± SD	conv. (%)	± SD	conv. (%)	± SD	conv. (%)	± SD	conv. (%)	± SD
**1**	1.1	0.1	2.7	0.1	15.3	0.7	47.2	1.0	33.7	1.5
**2**	0.5	0.0	4.5	0.2	19.3	0.1	56.9	3.9	18.9	3.8
**3**	76.3	0.5	2.2	0.3	4.0	0.1	13.5	0.4	4.0	0.2
**4**	1.8	2.4	4.2	0.1	18.5	0.2	62.7	2.1	12.9	0.3
**5**	0.2	0.1	5.6	0.9	19.0	0.9	37.5	1.7	37.7	1.5
**6**	0.1	0.0	3.4	0.2	17.5	0.7	57.1	0.4	21.9	0.9
**7**	1.5	0.3	5.5	1.8	21.2	0.6	70.5	1.3	1.2	2.2
**8**	0.5	0.0	4.1	0.4	20.0	1.9	75.4	1.5	0.0	0.0

HPLC analysis of Dm-dNK/EbPPK cascade reactions (9-12)

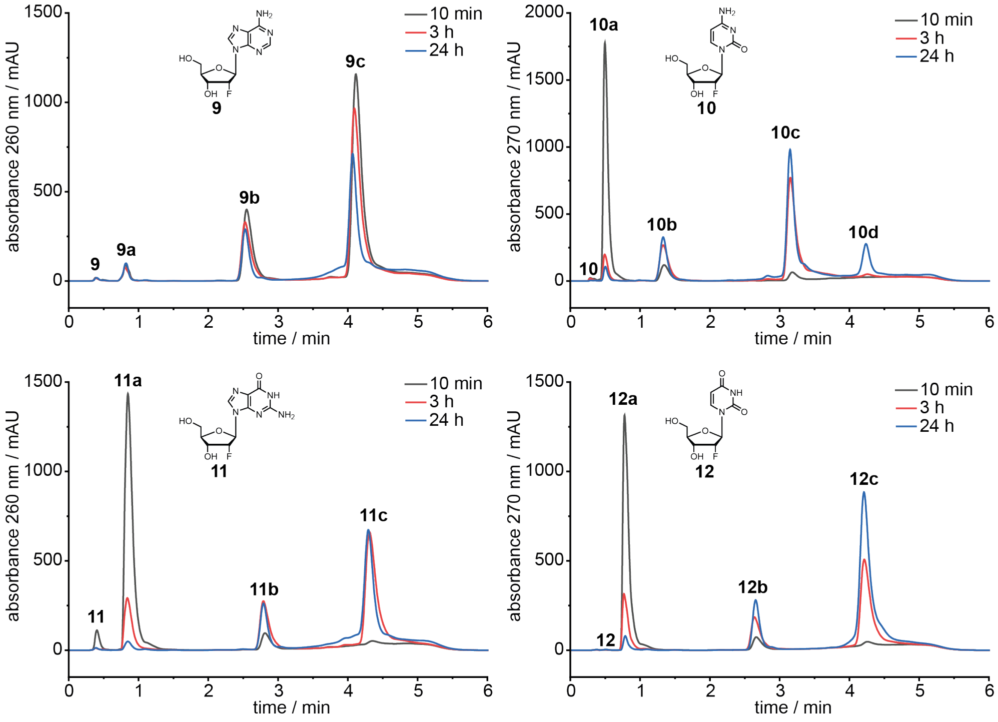

Fig. S15: Representative HPLC analysis of the Dm-dNK/EbPPK cascade reaction starting from 2′-fluoro-2′-deoxynucleosides (**9**-**12**).

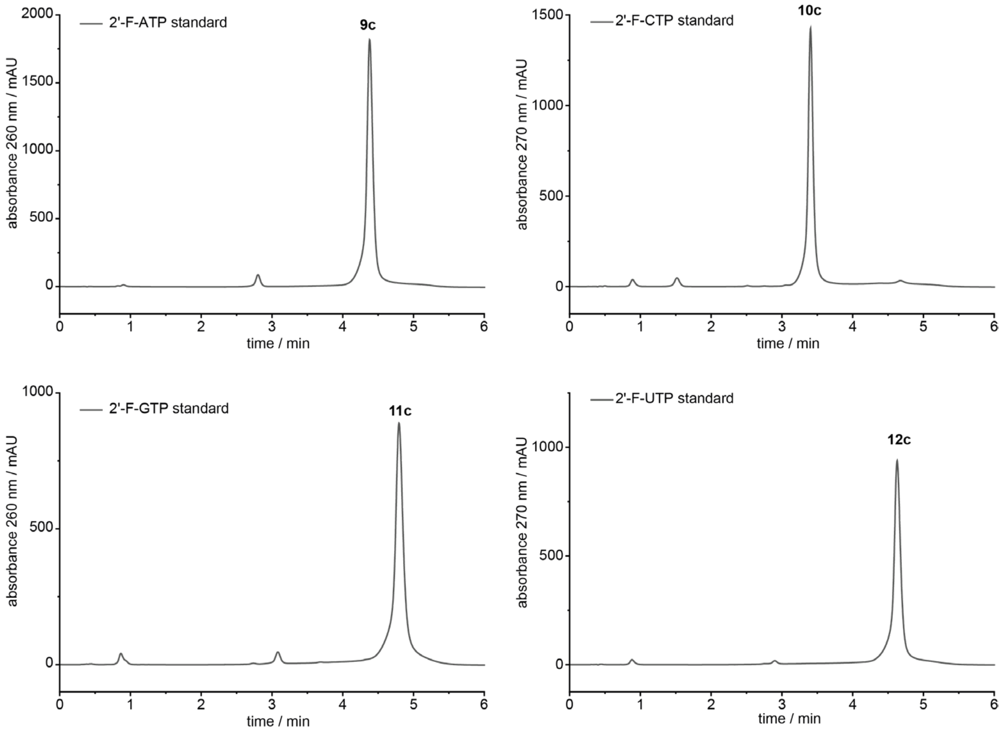

Fig. S16: HPLC analysis of commercial standards (Jena Bioscience) from compounds **9c**-**12c**.

HPLC analysis of Dm-dNK/EbPPK cascade reactions (13-17)

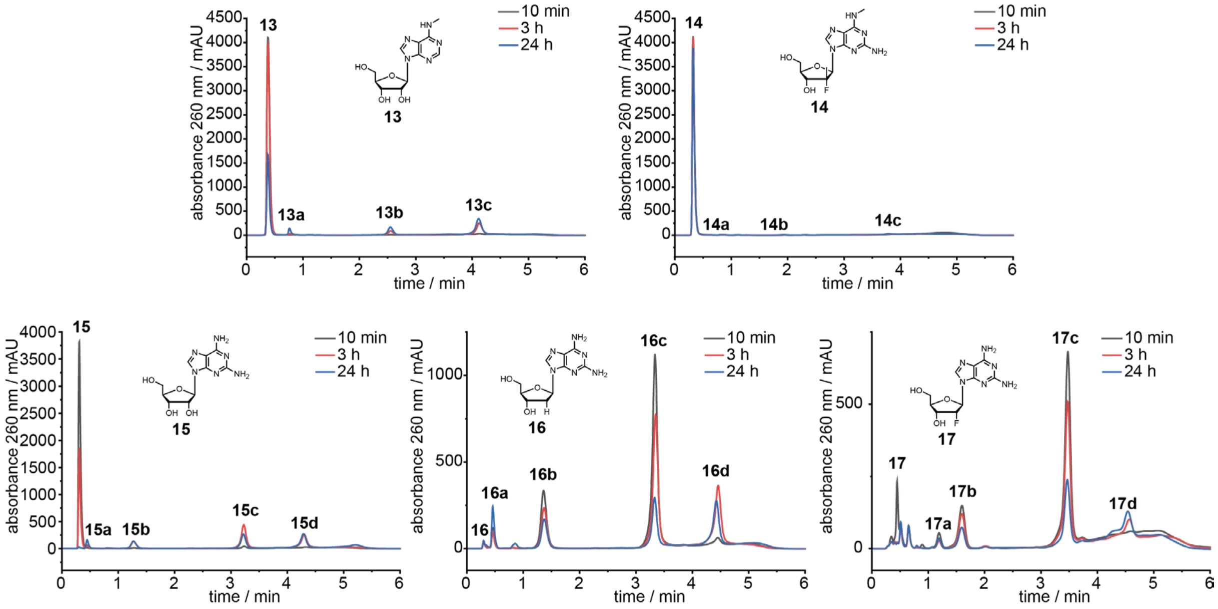

Fig. S17: Representative HPLC analysis of the Dm-dNK/EbPPK cascade reaction starting from modified nucleosides (**13**-**17**).

LC-TOF-MS analysis of Dm-dNK/EbPPK cascade reactions (9-12)

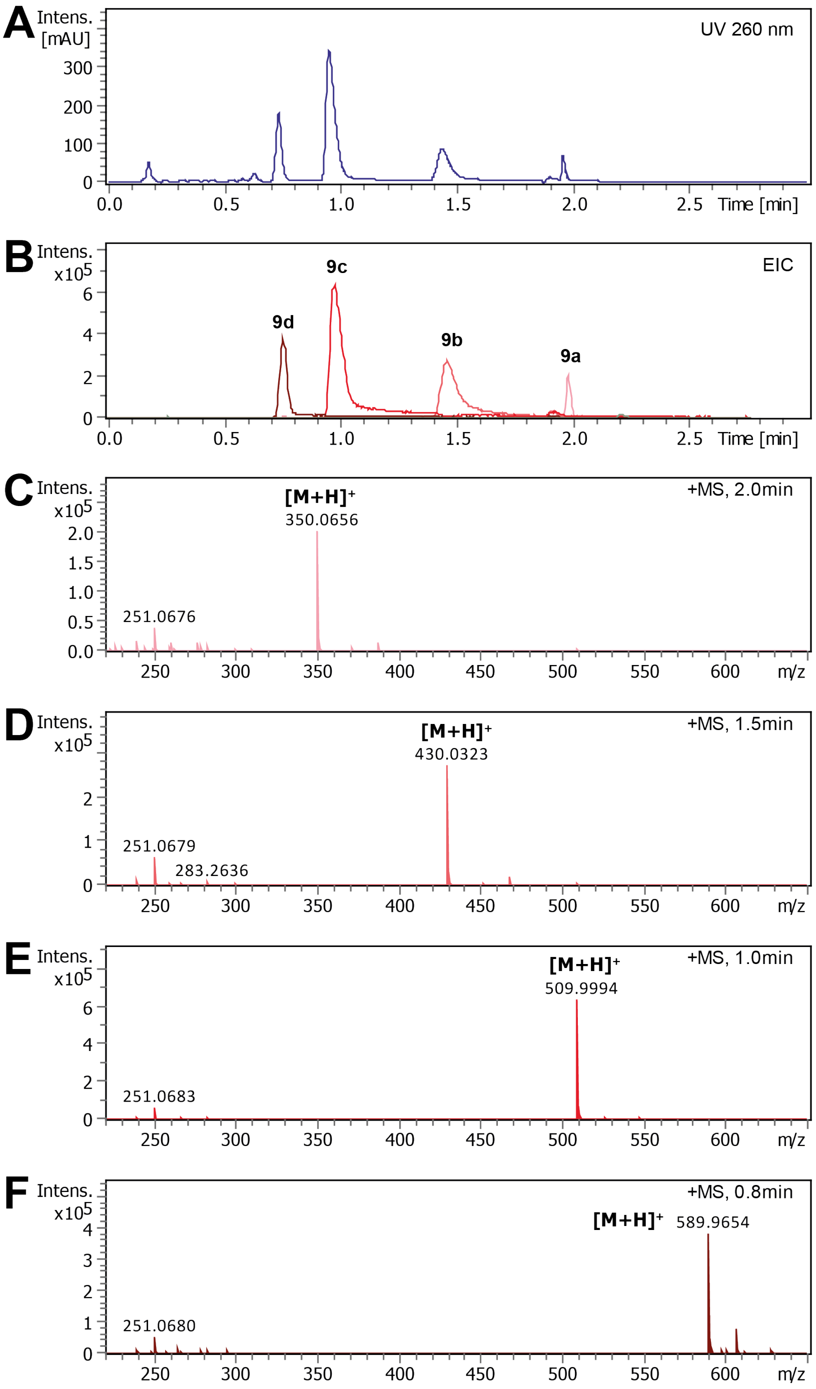

Fig. S18: LC-TOF-MS analysis of the Dm-dNK/EbPPK cascade reaction starting from **9**. A) UV chromatogram at 260 nm. B) Extracted-ion chromatogram for **9a-9d**. C)-F) Mass spectrum for **9a-9d**.

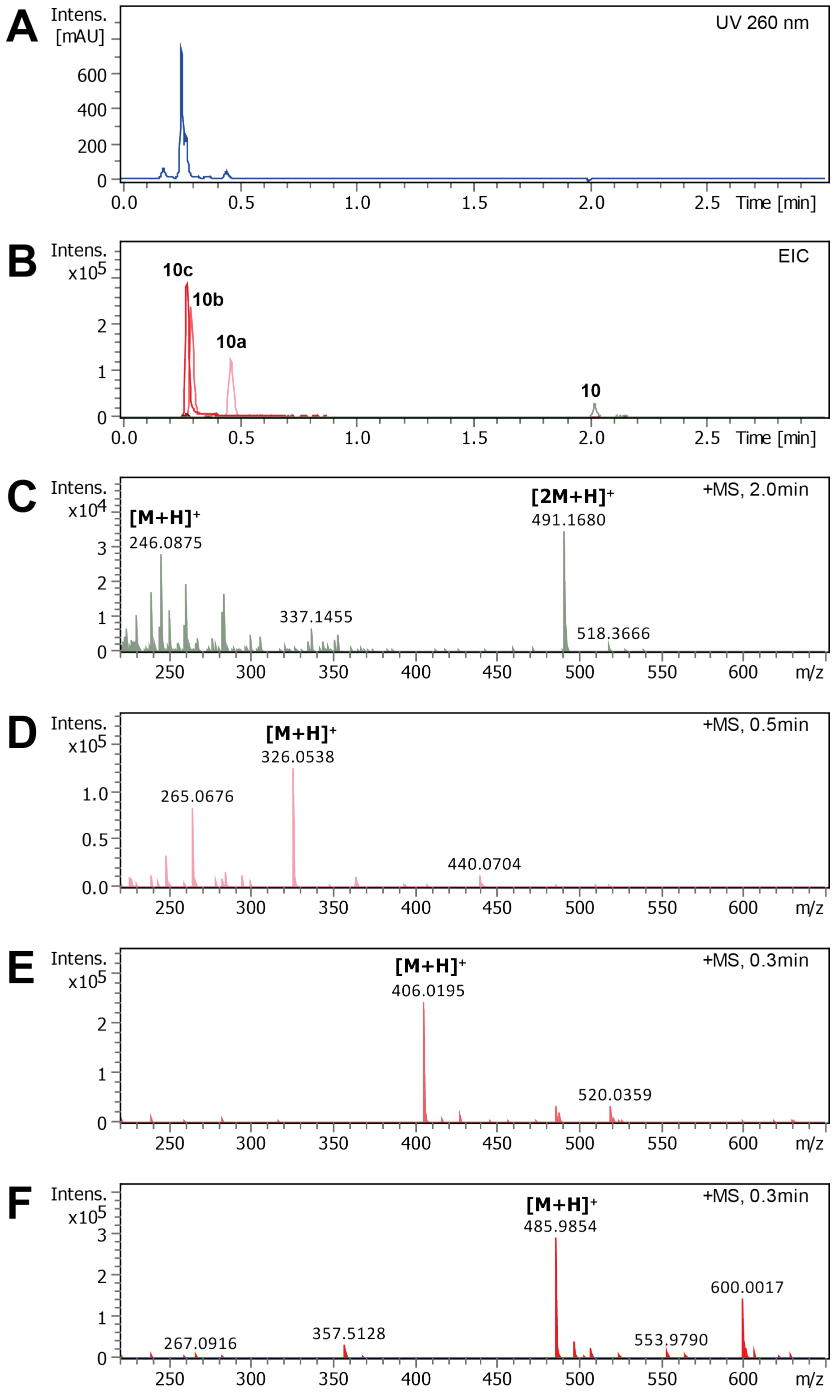

Fig. S19: LC-TOF-MS analysis of the Dm-dNK/EbPPK cascade reaction starting from **10**. A) UV chromatogram at 260 nm. B) Extracted-ion chromatogram for **10**-**10c**. C)-F) Mass spectrum for **10**-**10c**.

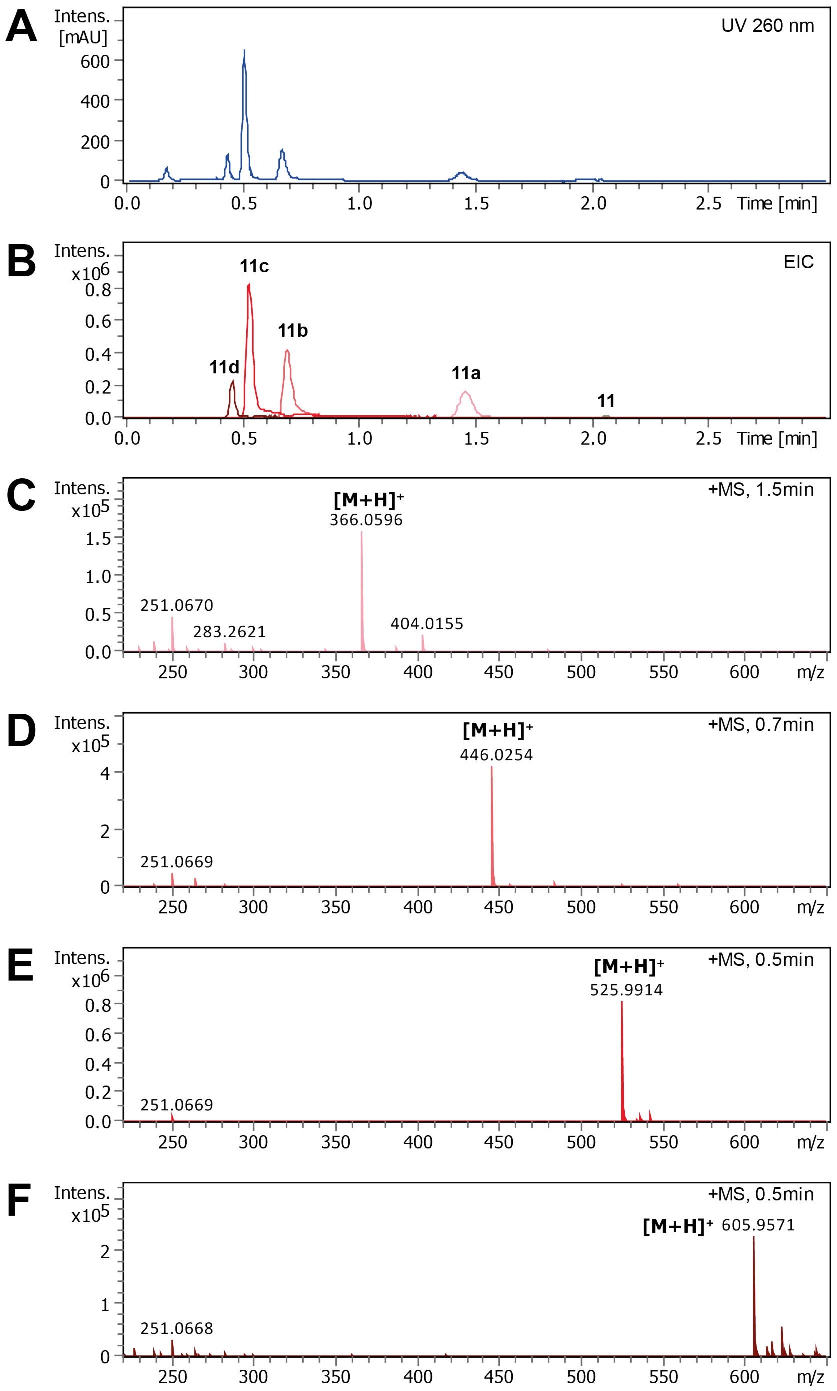

Fig. S20: LC-TOF-MS analysis of the Dm-dNK/EbPPK cascade reaction starting from **11**. A) UV chromatogram at 260 nm. B) Extracted-ion chromatogram for **11-11d**. C)-F) Mass spectrum for **11a-11d**.

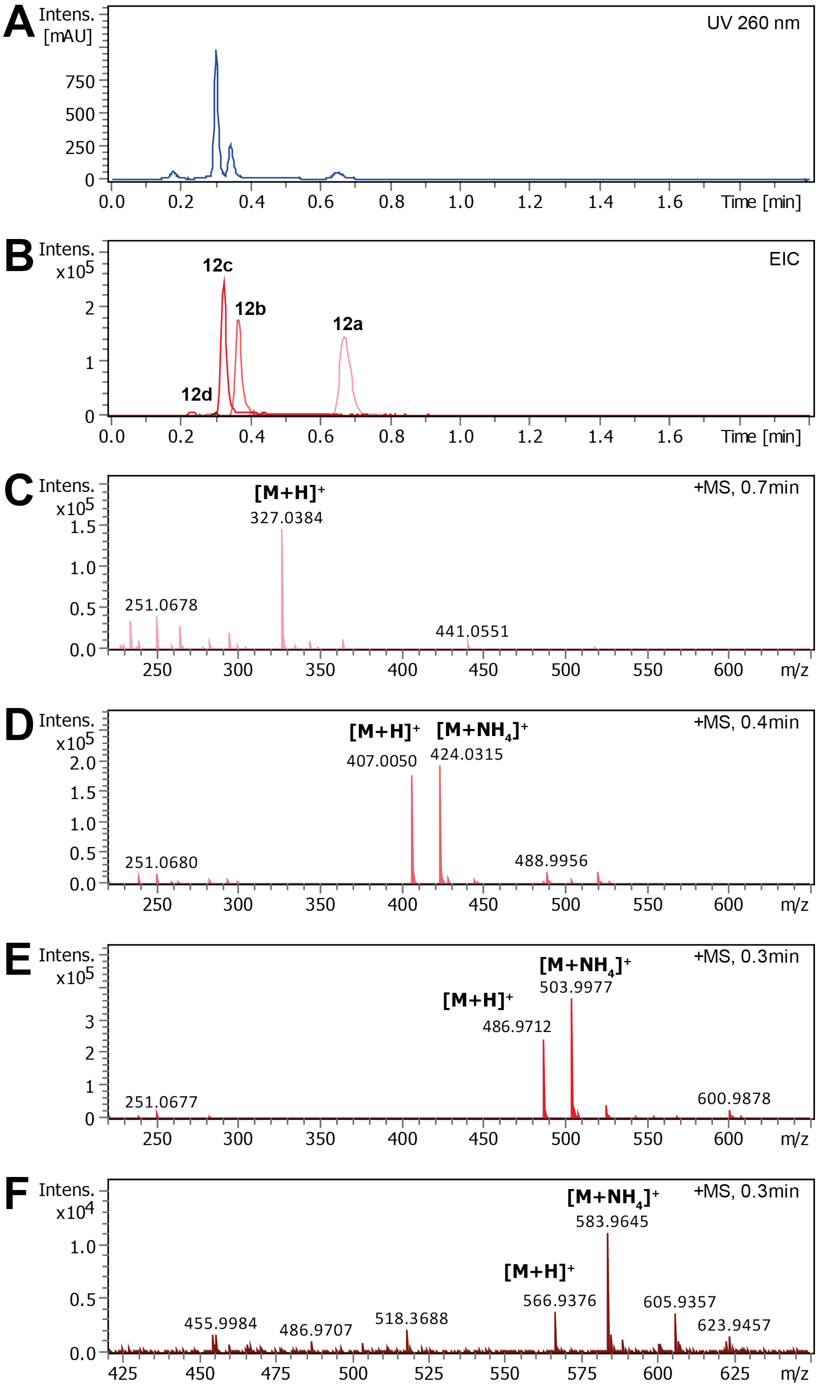

Fig. S21: LC-TOF-MS analysis of the Dm-dNK/EbPPK cascade reaction starting from **12**. A) UV chromatogram at 260 nm. B) Extracted-ion chromatogram for **12a**-**12d**. C)-F) Mass spectrum for **12a**-**12d**.

LC-TOF-MS analysis of Dm-dNK/EbPPK cascade reactions (13-17)

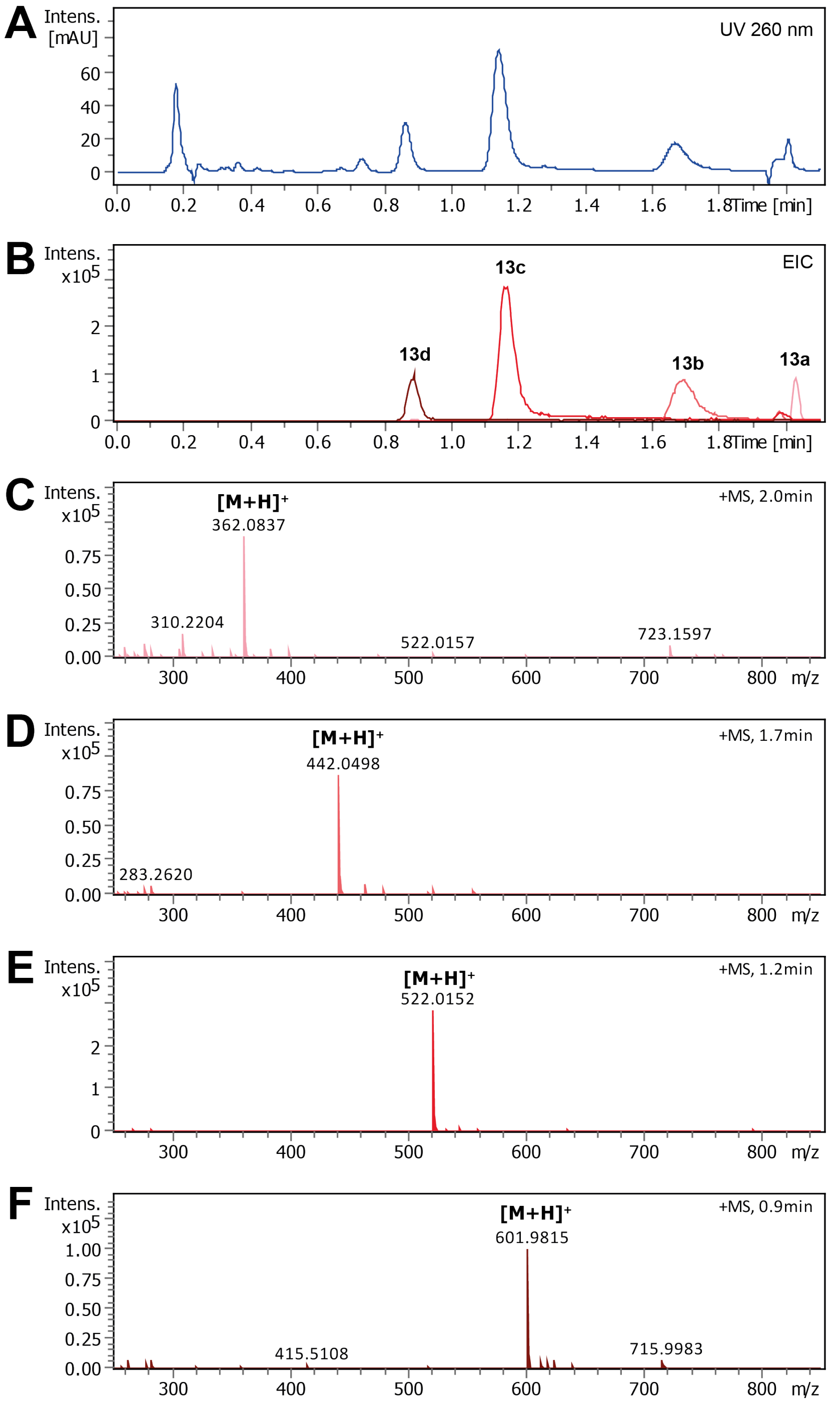

Fig. S22: LC-TOF-MS analysis of the Dm-dNK/EbPPK cascade reaction starting from **13**. A) UV chromatogram at 260 nm. B) Extracted-ion chromatogram for **13a-13d**. C)-F) Mass spectrum for **13a-13d**.

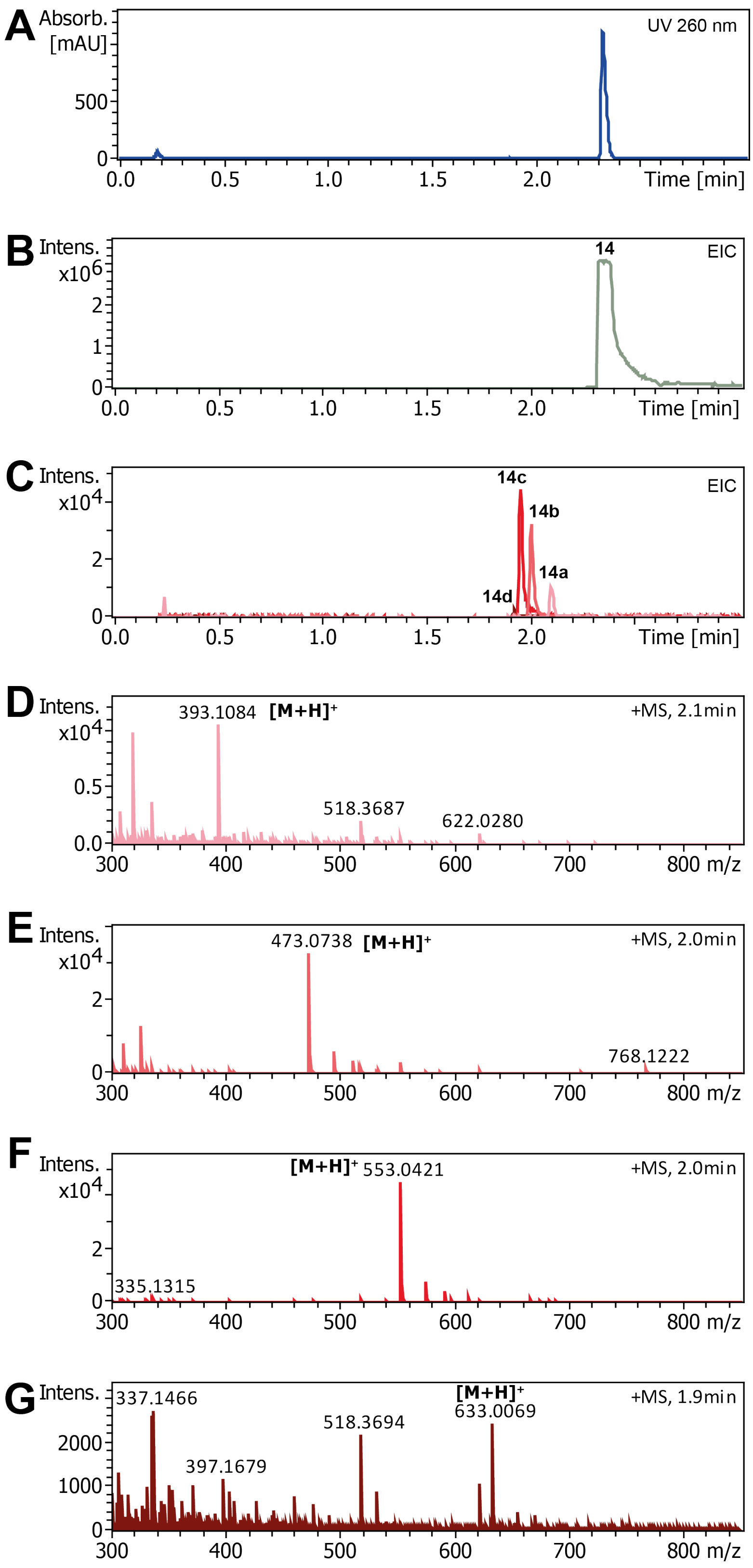

Fig. S23: LC-TOF-MS analysis of the Dm-dNK/EbPPK cascade reaction starting from **14**. A) UV chromatogram at 260 nm. B) Extracted-ion chromatogram for **14**. C) Extracted-ion chromatogram for **14a-14d**. D)-G) Mass spectrum for **14a-14d**.

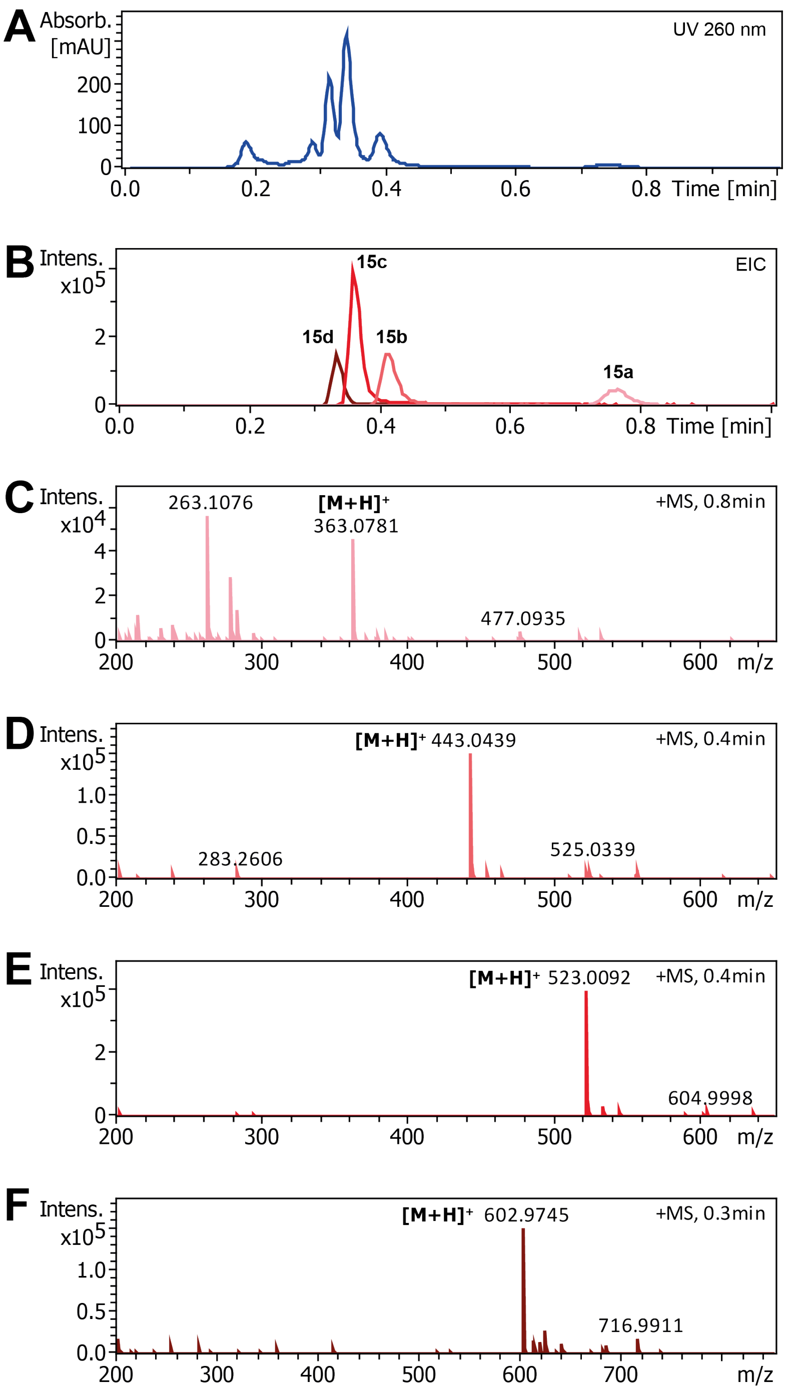

Fig. S24: LC-TOF-MS analysis of the Dm-dNK/EbPPK cascade reaction starting from **15**. A) UV chromatogram at 260 nm. B) Extracted-ion chromatogram for **15a-15d**. C)-F) Mass spectrum for **15a-15d**.

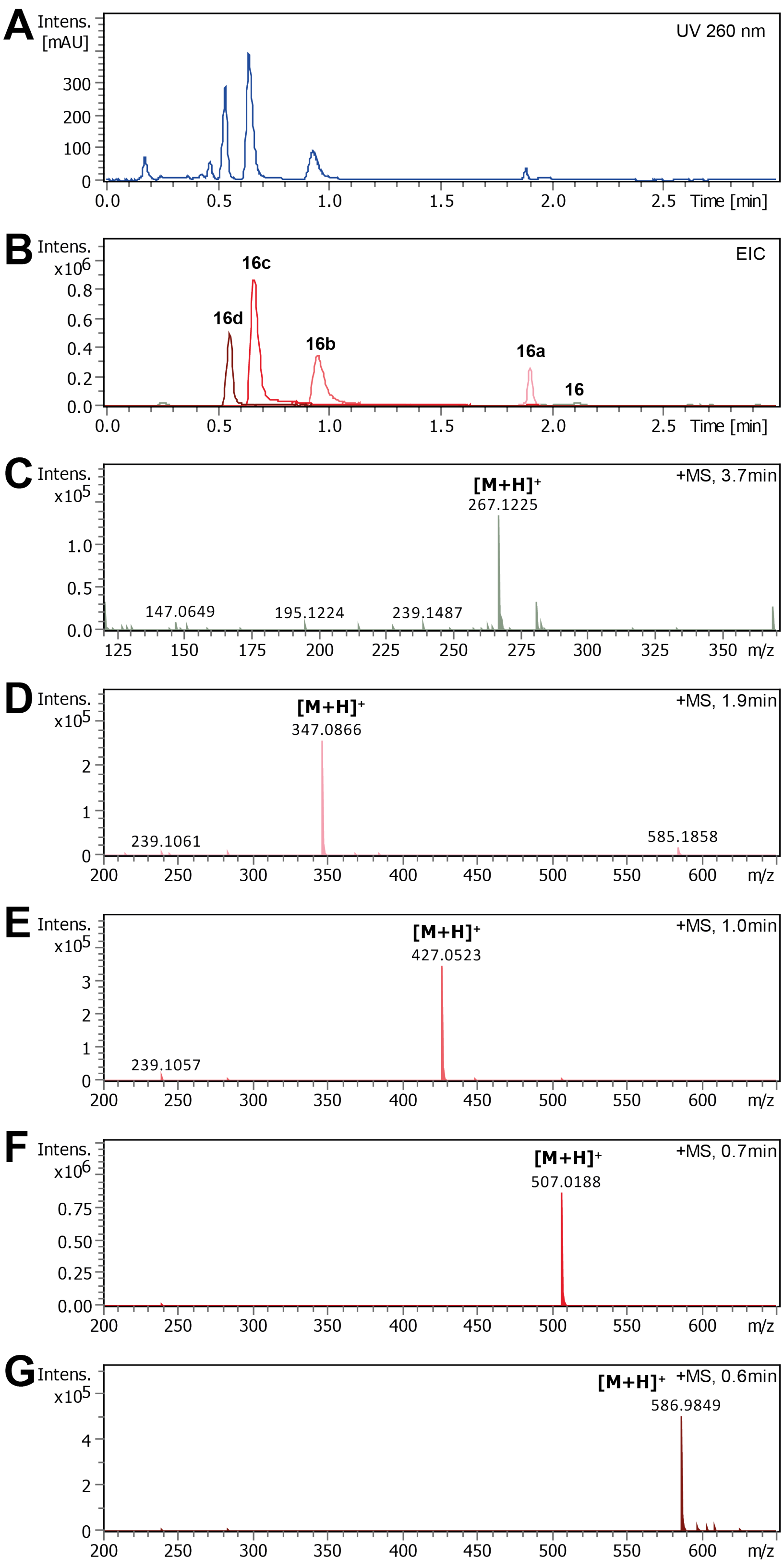

Fig. S25: LC-TOF-MS analysis of the Dm-dNK/EbPPK cascade reaction starting from **16**. A) UV chromatogram at 260 nm. B) Extracted-ion chromatogram for **16-16d**. C)-G) Mass spectrum for **16-16d**.

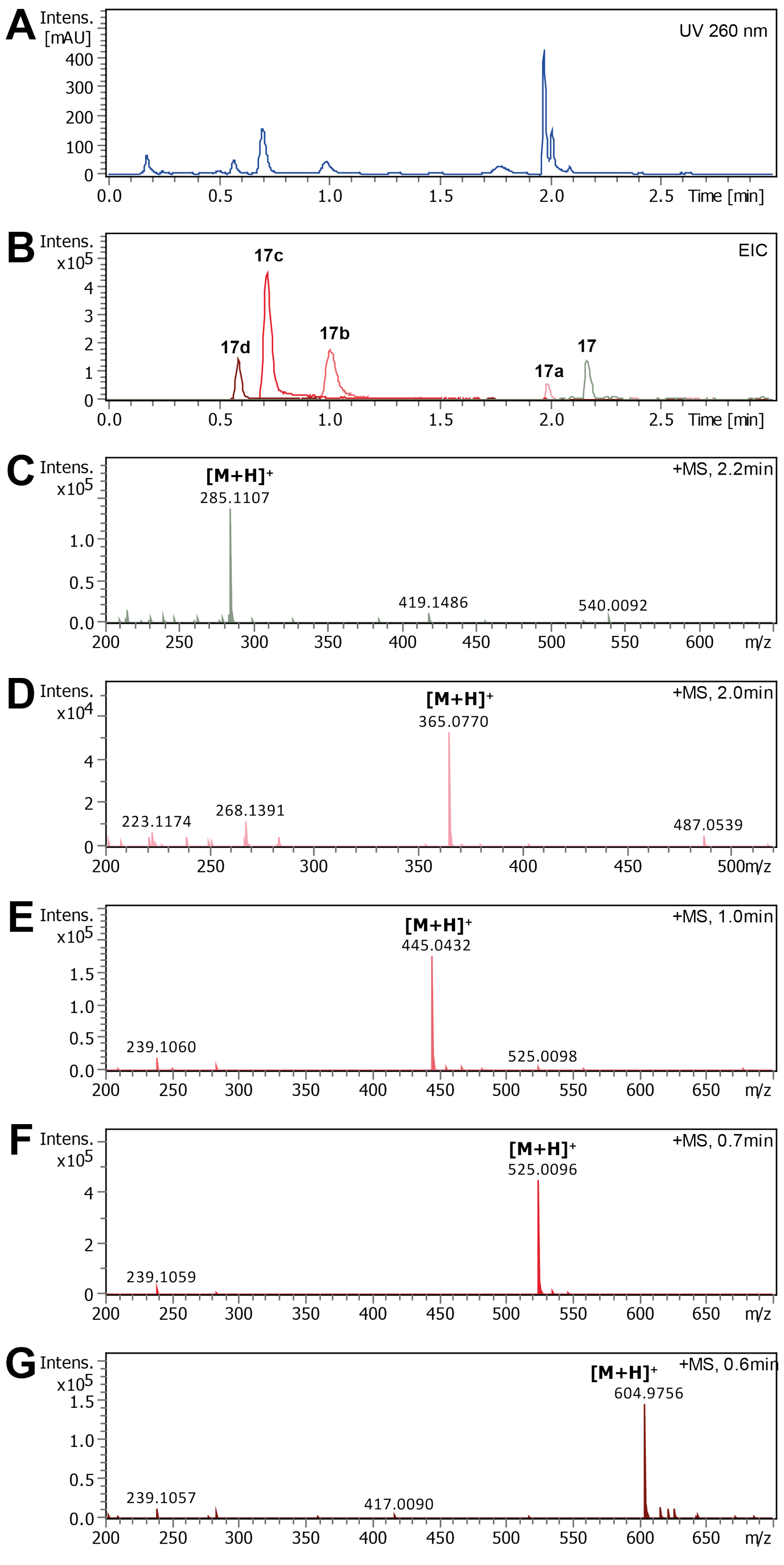

Fig. S26: LC-TOF-MS analysis of the Dm-dNK/EbPPK cascade reaction starting from **17**. A) UV chromatogram at 260 nm. B) Extracted-ion chromatogram for **17-17d**. C)-G) Mass spectrum for **17-17d**.

Table S3: Conversions of Dm-dNK/EbPPK cascades starting from 9-17. Average conversion and standard deviation of three independent experiments.

	nucleoside		**a** (NMP)		**b** (NDP)		**c** (NTP)		**d** (N4P)	
**10 min**	conv. (%)	± SD	conv. (%)	± SD	conv. (%)	± SD	conv. (%)	± SD	conv. (%)	± SD
**9**	0.6	0.1	3.4	0.1	24.8	0.9	71.2	1.0	0.0	0.0
**10**	1.2	0.0	85.1	1.8	9.8	1.3	3.9	0.5	0.0	0.0
**11**	4.9	1.3	88.2	0.5	5.9	0.7	1.1	0.1	0.0	0.0
**12**	0.1	0.0	84.2	10.4	7.4	0.5	8.3	10.4	0.0	0.0
**13**	98,5	0,3	0,3	0,0	0,4	0,0	0,9	0,3	0,0	0,0
**14**	99,0	0,4	0,8	0,3	0,0	0,0	0,2	0,1	0,0	0,0
**15**	95,0	0,2	0,7	0,1	1,3	0,2	2,5	0,2	0,5	0,1
**16**	1,1	0,0	3,6	0,1	22,9	0,4	69,6	0,4	2,8	0,5
**17**	12,8	0,4	4,3	0,0	17,4	0,7	65,3	0,5	0,2	0,3

	nucleoside		**a** (NMP)		**b** (NDP)		**c** (NTP)		**d** (N4P)	
**3 h**	conv. (%)	± SD	conv. (%)	± SD	conv. (%)	± SD	conv. (%)	± SD	conv. (%)	± SD
**9**	0.5	0.1	3.6	0.2	25.1	0.5	70.7	0.7	0.0	0.0
**10**	0.8	0.0	10.7	2.6	25.0	0.7	62.1	3.0	1.4	0.2
**11**	0.6	0.2	22.1	4.9	21.9	0.4	55.5	4.4	0.0	0.0
**12**	0.1	0.0	18.6	4.3	21.4	0.6	60.0	3.7	0.0	0.0
**13**	85,6	1,1	0,9	0,1	3,7	0,4	9,8	0,6	0,0	85,6
**14**	98,6	0,3	0,8	0,3	0,2	0,0	0,3	0,1	0,0	98,6
**15**	43,6	1,5	2,2	0,1	9,4	0,4	28,1	1,0	16,6	43,6
**16**	1,2	0,0	3,3	0,1	17,6	0,3	51,4	0,1	26,5	1,2
**17**	5,0	0,9	3,1	0,4	20,4	1,7	61,8	1,5	9,8	5,0

	nucleoside		**a** (NMP)		**b** (NDP)		**c** (NTP)		**d** (N4P)	
**24 h**	conv. (%)	± SD	conv. (%)	± SD	conv. (%)	± SD	conv. (%)	± SD	conv. (%)	± SD
**9**	1.2	0.1	6.8	0.7	28.9	2.3	63.2	3.2	0.0	0.0
**10**	0.2	0.2	4.2	0.2	22.3	1.3	58.7	1.4	14.5	2.3
**11**	0.8	0.0	4.9	0.3	25.2	1.3	69.1	1.5	0.0	0.0
**12**	0.1	0.0	4.5	0.4	22.6	2.3	72.7	2.7	0.0	0.0
**13**	51,5	3,7	5,3	0,2	11,5	0,8	23,7	0,3	7,9	51,5
**14**	98,0	0,2	0,6	0,3	0,6	0,1	0,8	0,1	0,0	98,0
**15**	2,4	0,2	9,8	0,8	18,6	1,1	33,4	0,8	35,9	2,4
**16**	2,3	0,3	12,3	0,8	20,8	0,7	30,7	0,8	33,9	2,3
**17**	8,2	0,4	6,0	0,7	20,4	2,6	42,5	2,8	23,0	8,2

HPLC analysis of Dm-dNK/EbPPK cascade reactions (18-19)

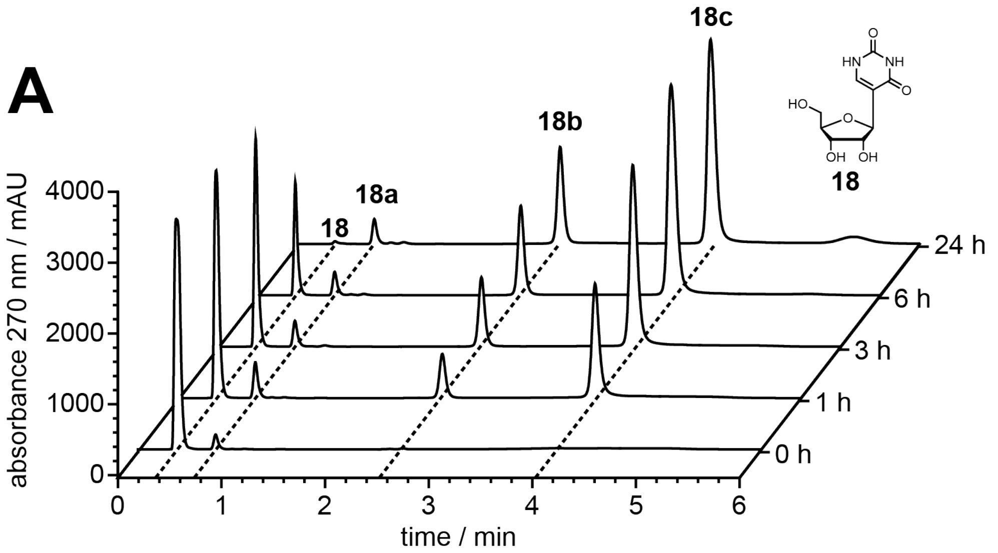

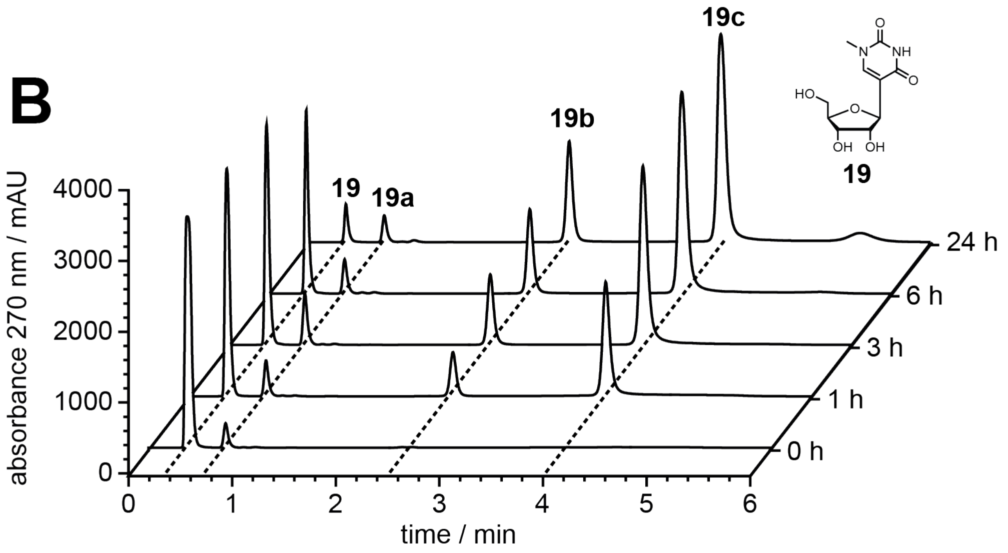

Fig. S27: Representative HPLC analysis of the Dm-dNK/EbPPK cascade reaction starting from A) pseudouridine (**18**) or B) *N*1-methylpseudouridine (**19**).

LC-TOF-MS analysis of Dm-dNK/EbPPK cascade reactions (18-19)

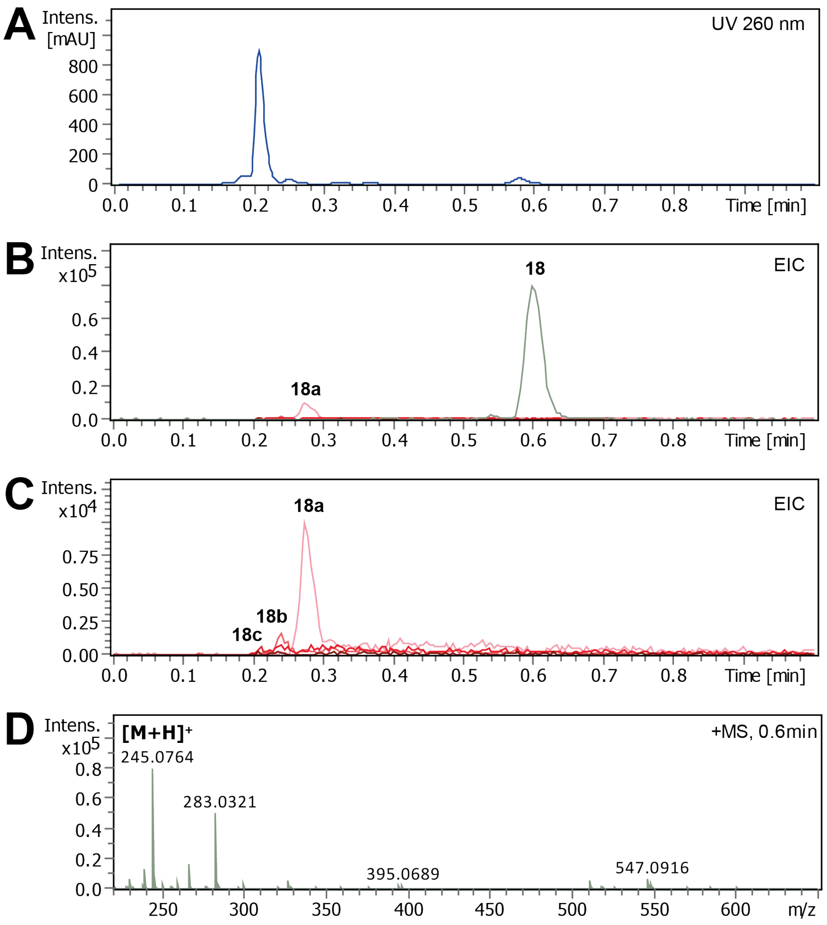

Fig. S28: LC-TOF-MS analysis of the Dm-dNK/EbPPK cascade reaction starting from **18**. A) UV chromatogram at 260 nm. B) Extracted-ion chromatogram for **18-18a**. C) Extracted-ion chromatogram for **18a-18c**. D) Mass spectrum for **18a**.

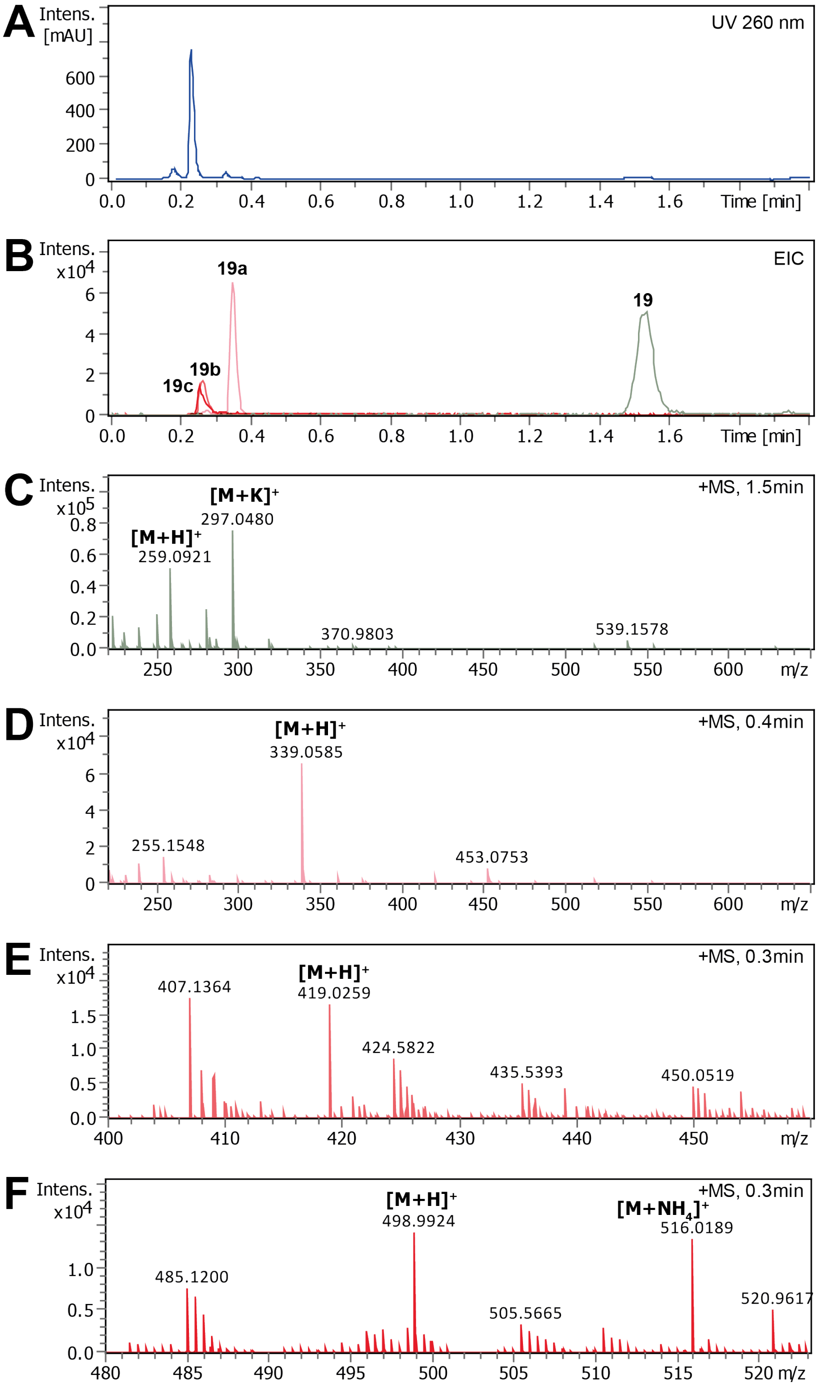

Fig. S29: LC-TOF-MS analysis of the Dm-dNK/EbPPK cascade reaction starting from **19**. A) UV chromatogram at 260 nm. B) Extracted-ion chromatogram for **19-19c** C)-F) Mass spectrum for **19-19c**.

Table S4: Conversions of Dm-dNK/EbPPK cascades starting from 18-19 (5 mM).

Average conversion and standard deviation of three independent experiments.

	nucleoside		**a** (NMP)		**b** (NDP)		**c** (NTP)		**d** (N4P)	
**0 h**	conv. (%)	± SD	conv. (%)	± SD	conv. (%)	± SD	conv. (%)	± SD	conv. (%)	± SD
**18**	95.1	0.7	4.4	0.6	0.5	0.1	0.0	0.0	0.0	0.0
**19**	93.0	0.7	6.6	0.7	0.4	0.0	0.0	0.0	0.0	0.0

	nucleoside		**a** (NMP)		**b** (NDP)		**c** (NTP)		**d** (N4P)	
**1 h**	conv. (%)	± SD	conv. (%)	± SD	conv. (%)	± SD	conv. (%)	± SD	conv. (%)	± SD
**18**	51.1	3.8	6.4	0.8	11.0	0.7	31.5	2.4	0.0	0.0
**19**	45.6	3.4	18.3	2.5	10.2	0.6	25.9	0.5	0.0	0.0

	nucleoside		**a** (NMP)		**b** (NDP)		**c** (NTP)		**d** (N4P)	
**3 h**	conv. (%)	± SD	conv. (%)	± SD	conv. (%)	± SD	conv. (%)	± SD	conv. (%)	± SD
**18**	31.8	2.4	4.3	0.3	15.3	0.5	48.5	1.7	0.0	0.0
**19**	33.9	2.5	7.1	0.9	14.3	0.6	44.6	1.3	0.0	0.0

	nucleoside		**a** (NMP)		**b** (NDP)		**c** (NTP)		**d** (N4P)	
**6 h**	conv. (%)	± SD	conv. (%)	± SD	conv. (%)	± SD	conv. (%)	± SD	conv. (%)	± SD
**18**	15.1	1.3	4.0	0.2	19.3	0.6	61.3	1.0	0.2	0.4
**19**	22.6	1.5	5.0	0.4	17.0	0.4	54.9	1.5	0.5	0.4

	nucleoside		**a** (NMP)		**b** (NDP)		**c** (NTP)		**d** (N4P)	
**24 h**	conv. (%)	± SD	conv. (%)	± SD	conv. (%)	± SD	conv. (%)	± SD	conv. (%)	± SD
**18**	0.6	0.1	5.2	0.1	24.5	0.5	66.9	3.3	2.7	3.6
**19**	4.2	0.8	4.5	0.3	22.3	0.6	64.1	3.2	4.8	2.6

Preparative separation of 19a-19d.

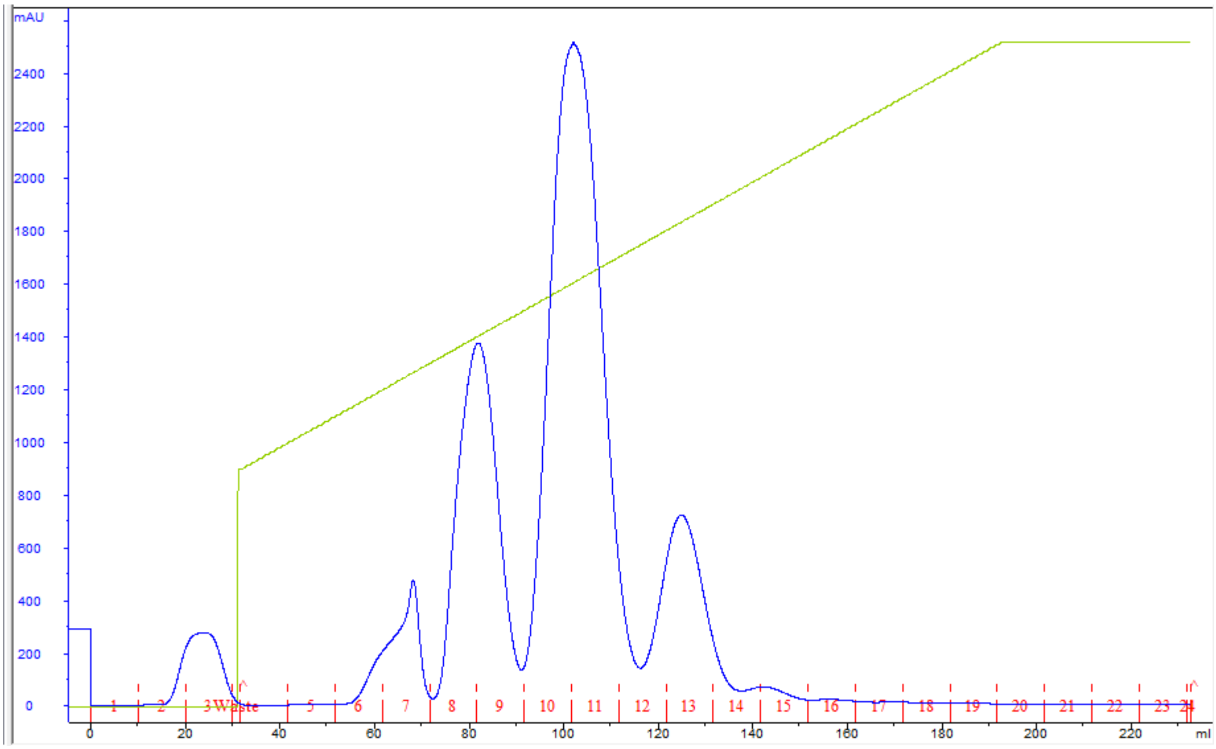

Fig. S30: Preparative anion exchange separation of **19a**-**19d**, from 2 mL Dm-dNK/EbPPK reaction. Fractions 6-7 contain m^{1}ΨMP (**19a**), fractions 8-9 m^{1}ΨDP (**19b**), fractions 10-11 m^{1}ΨTP (**19c**) and fractions 13-14 m^{1}Ψ4P (**19d**).

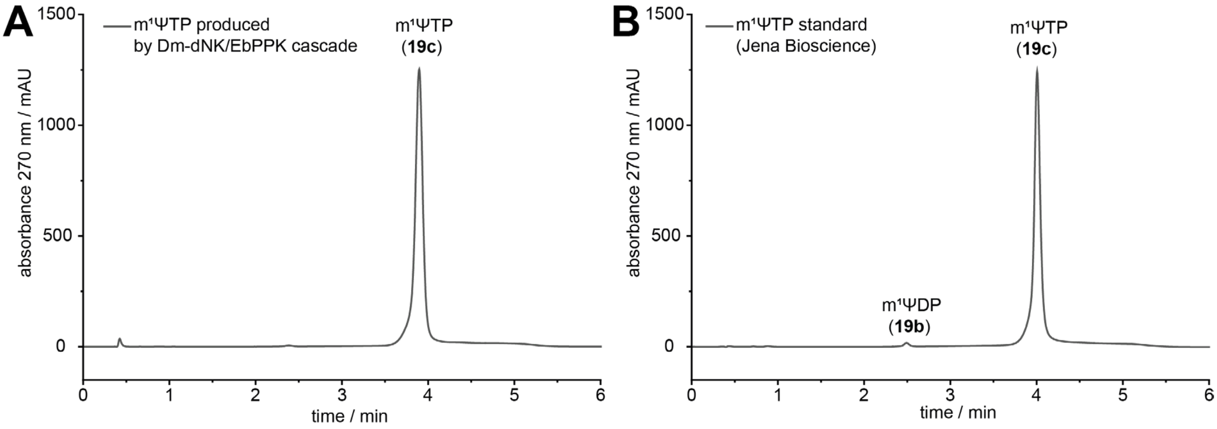

Fig. S31: HPLC analysis of the Dm-dNK/EbPPK cascade reaction with preparative amounts (5 mM, 2 mL) of **19** after work up (ÄKTA purification, lyophilisation, precipitation with acetone and redissolving in ddH_2O) (A) and of a commercial m^{1}ΨTP standard (Jena Bioscience) (B).

References

1. Mitton-Fry, R. M.; Eschenbach, J.; Schepers, H.; Rasche, R.; Erguven, M.; Kümmel, D.; Rentmeister, A.; Cornelissen, N. V., Chemo-enzymatic production of base-modified ATP analogues for polyadenylation of RNA. *Chem. Sci.* **2024,** *15* (32), 13068-13073.

2. Mitton-Fry, R. M.; Rasche, R.; Lawrence-Dörner, A. M.; Eschenbach, J.; Tekath, A.; Rentmeister, A.; Kümmel, D.; Cornelissen, N. V., Structure-guided engineering of a polyphosphate kinase 2 class III from an Erysipelotrichaceae bacterium to produce base-modified purine nucleotides. *RSC Chem Biol* **2025,** *6* (8), 1328-1335.

3. Christ, J. J.; Willbold, S.; Blank, L. M., Polyphosphate Chain Length Determination in the Range of Two to Several Hundred P-Subunits with a New Enzyme Assay and (31)P NMR. *Anal Chem* **2019,** *91* (12), 7654-7661.
